# Spatial and Temporal Shifts of Endophytic Bacteria in Conifer Seedlings of *Abies religiosa* (Kunth) Schltdl. & Cham.

**DOI:** 10.1007/s00248-024-02398-9

**Published:** 2024-07-03

**Authors:** Luc Dendooven, Valentín Pérez-Hernández, Gabriel Navarro-Pérez, Juanita Tlalmis-Corona, Yendi E. Navarro-Noya

**Affiliations:** 1 Laboratory of Soil Ecology, Cinvestav, Mexico City, Mexico; 2 Laboratorio de Interacciones Bióticas, Centro de Investigación en Ciencias Biológicas, Universidad Autónoma de Tlaxcala, San Felipe Ixtacuixtla, Mexico

**Keywords:** High mountain ecosystems, Tree microbiome, Forest conservation, Plant-microbe interaction, Plant microbiome

## Abstract

Endophytes play an important role in plant development, survival, and establishment, but their temporal dynamics in young conifer plants are still largely unknown. In this study, the bacterial community was determined by metabarcoding of the 16S rRNA gene in the rhizoplane, roots, and aerial parts of 1- and 5-month-old seedlings of natural populations of *Abies religiosa* (Kunth) Schltdl. & Cham. In 1-month-old seedlings, *Pseudomonas* dominated aerial parts (relative abundance 71.6%) and roots (37.9%). However, the roots exhibited significantly higher bacterial species richness than the aerial parts, with the dissimilarity between these plant sections mostly explained by the loss of bacterial amplification sequence variants. After 5 months, *Mucilaginibacter* dominated in the rhizoplane (9.0%), *Streptomyces* in the roots (12.2%), and *Pseudomonas* in the aerial parts (18.1%). The bacterial richness and community structure differed significantly between the plant sections, and these variations were explained mostly by 1-for-1 substitution. The relative abundance of putative metabolic pathways significantly differed between the plant sections at both 1 and 5 months. All the dominant bacterial genera (e.g., *Pseudomonas* and *Burkholderia-Caballeronia-Paraburkholderia*) have been reported to have plant growth-promoting capacities and/or antagonism against pathogens, but what defines their role for plant development has still to be determined. This investigation improves our understanding of the early plant-bacteria interactions essential for natural regeneration of *A. religiosa* forest.

**Supplementary Information:**

The online version contains supplementary material available at 10.1007/s00248-024-02398-9.

## Introduction

Plants create internally an environment or ecosystem with very specific characteristics. This environment contains many microorganisms with a genetic diversity that is often larger than that of their host [1, 2]. These microorganisms, also called endophytes, are essential for the well-being of their host although some of them can become pathogens [3]. Plant tissue, host type and age, and environmental factors determine the endophytic microbial structure and their metabolic functions [4].

The largest proportion of plant endophytic microorganisms are bacteria, mostly members of Pseudomonadota (formerly Proteobacteria [5]) and Actinomycetota (formerly Actinobacteriota [5]) [2]. These endophytic bacteria can fix nitrogen for the plants, e.g., *Rhizobium*, liberate nutrients, such as potassium and minor elements, mineralize organic nitrogen and phosphorus, be antagonistic against pathogens, or produce metabolites that stimulate plant growth [6]. As such, they mitigate stress and promote plant growth.

Bacteria in plants come initially from the seeds, while bacterial colonization usually starts at the root surface. After successful entry, bacteria can move to the aerial parts by the transpiration stream and with the support of bacterial flagella [7]. Root exudates, e.g., organic acids, amino acids, and proteins, can participate in the recruitment of bacterial endophytes from the soil or substrate in which they grow [8]. Root exudates probably contain substrates that initiate early communication between host plants and bacterial endophytes and consequently drive the colonization process. The composition of these exudates changes at different life stages of the host plant, and the composition and diversity of endophytic bacteria also change [9]. The bacterial composition of the native soil is also important for the recruitment of bacterial endophytes by the host plant. For example, the root endophyte community composition of *Arabidopsis* Heynh. in Holl & Heynh. plants grown in different soils was different, which may indicate that the soil represents an important source of environmental inoculum for plants [10]. Overall, much of our understanding of plant-microbe interactions originates from studies on model or agricultural plants, while investigation into tree microbiomes remains unexplored [11].

High mountain temperate forests are important ecosystems as they prevent erosion, maintain soil quality, sequester C, and are home to highly diverse life forms [12]. These valuable ecosystems are under threat due to land-use change and the increase in the frequency and severity of drought and heat stress associated with climate change [13, 14]. In Mexico, more than 50% of temperate forest coverage has been lost, fragmented, or degraded. *Abies religiosa* (Kunth) Schltdl. & Cham. (fir) is an important species in the coniferous ecosystem of the high mountains of Mexico, and it is the overwintering host of the Monarch butterfly (*Danaus plexippus* L.) [15]. However, *A. religiosa* is one of the most affected forest trees due to habitat loss and overexploitation [16]. It is used as an ornamental tree at Christmas and for its wood [17]. If adequate actions are not implemented soon to promote the conservation of *A. religiosa*, there is a risk of local extinction in some parts of Mexico.

Seedling recruitment is important to the survival of *A. religiosa*, as well as all conifers, given their susceptibility to environmental stressors and pathogens. The seedling microbiome might play an important role in their survival. Understanding endophyte community assembly during seedling establishment and survival of seedlings may be key to designing effective conservation strategies. Insights into temporal changes in the microbiome could guide management practices to maximize the survival and growth rates of *A. religiosa*. What changes occur when the *A. religiosa* plantlets grow is still largely unknown. In this study, we investigated the bacterial endophytes of *A. religiosa* 1- and 5-month-old seedlings growing on the flanks of the volcano in “La Malinche” national park. The objectives were (i) to compare the bacterial community and their putative metabolic functions in the aerial parts of *A. religiosa* seedlings with that in the roots after 1 month; (ii) to compare the bacterial community and their putative metabolic functions in the aerial parts, roots, and rhizoplane after 5 months; and (iii) to compare the bacterial community and their putative metabolic functions in the roots and aerial parts after 1 and 5 months (i.e., changes over time).

## Materials and Methods

### Sampling Site and Soil Characterization

The sampling area was in the “La Malinche” National Park bordering the states of Tlaxcala and Puebla (Mexico). *Abies religiosa* patches in La Malinche mountain are confined within intricate ravines where the microclimate favors the growth of the tree. The seedlings were collected from locations at 3750 and 3900 m above sea level (a.s.l.) (Fig. S1). *Abies religiosa* seedlings were taken from six 4-m^2^ quadrants (N 19°14′3.4″ W 98°01′17.1″; N 19°14.4′2.8″ W 98°01′18.3″; N 19°14′43.5″ W 98°01′15.7″ at 3700 and (N 19°14′24.9″ W 98°01′27.5″; N 19°14′27.11″ W 98°01′36.66″; N 19°14′25.66″ W 98°01′28.91″ at 3900 m a.s.l.) after approximately 1 (November 17, 2015) and 5 months (March 30, 2016) after emergence. One-month-old seedlings (< 10 cm) had not yet reached the mineral soil and grew in the thick layer of moss, so the bacterial community was only studied in the roots and aerial part of the seedlings. After 5 months, the seedlings (ca. 15 cm) had reached the mineral soil, but they were still too small to form a well-developed rhizosphere, so the bacteria adhered to them (i.e., the rhizoplane) were collected.

The seedlings were carefully removed from the substrate with disinfected forceps, placed in sterile containers on ice, and transported to the laboratory for extraction of DNA. In the same quadrants, the organic layer was removed, and the soil was sampled and characterized. More details on the sampling design can be found in the Supplementary Figure S1. A total of 54 seedlings were sampled, 27 at each time point.

The pH was measured by mixing a soil sample with distilled water in a 1:2.5 ratio, and pH was determined with a potentiometer Mettler Toledo® Model S220 (New York, USA) [18], while a saturated soil sample was prepared with distilled water to determine the electrolytic conductivity (EC) with a conductometer Mettler Toledo® Model S220 (New York, USA) [19]. The soil particle size distribution was determined by the hydrometer method [20], while total C was determined with a Thermo Scientific™ FlashSmart™ Elemental Analyzer (Waltham, MA, USA) following the standard protocol given by the manufacturer. The inorganic C in soil was determined by adding 20 ml 1 M HCl solution to 2 g air-dried soil and trapping CO_2_ evolved in 20 ml 1 M NaOH, and the organic C was defined as the difference between the total and inorganic C. Total N was measured by the Kjeldahl method using concentrated H_2_SO_4_, K_2_SO_4_, and HgO to digest the sample [21]. The water holding capacity (WHC) was measured on water-saturated soil samples in a funnel and left to stand overnight. The weight of the water retained by the soil was considered the WHC.

### DNA Extraction, Amplification, and Sequencing

Endophytic bacterial DNA was isolated separately from the roots and aerial parts of each sampled seedling. First, seedlings were washed with tap water and then with sterile distilled water. Tissues were surface disinfected by serially immersing in commercial bleach 2.5% (v/v) for 5 min (roots for 15 min) and ethanol 70% (v/v) for 5 min and rinsed four times with sterile distilled water. The disinfection and elimination of epiphytic bacteria were confirmed with the absence of amplification of the 16S rRNA using water for the final wash as a template. After disinfection, the plants were separated into roots and aerial parts using sterile scissors and placed in 15-mL propylene tubes. Liquid nitrogen was applied to ~ 50 mg of plant tissue and ground with sterile beads on a FastPrep-24 (MP-Biomedicals, Santa Ana, CA). The ground tissues were added with 2 mL 10 mM Tris-HCl (pH 8) plus 160 μL 10 mg mL^−1^ lysozyme. The mixture was kept at 37°C for 1 h. The cells were lysed with 2 mL sodium dodecyl sulfate (SDS) 10% (w/v) [22]. Sterile glass beads were added and mixed on a vortex for 10 min. Proteins, SDS, and RNA were removed from the supernatant with EDTA (100 mM, pH 8.0) and 1/10 volume potassium acetate (5 M, pH 5.5) [23]. Samples were incubated on ice for 20 min and centrifuged at 10,000 *g* at 4 °C for 5 min to remove the precipitate. DNA was purified by extracting twice with 500 μL chloroform-isoamyl alcohol (24:1) and finally precipitated with ethanol.

The separated roots were placed in 15-mL propylene tubes to collect samples from the rhizoplane of 5-month-old seedlings. They were then rinsed twice with 5 mL of sterile distilled water, and the rhizoplane cells were obtained by shaking the roots at 300 rpm with 5 mL of sterile phosphate-buffered saline (PBS) pH 7.4 for 30 min. Subsequently, the roots were carefully removed from the tube using sterile forceps and disinfected following the previously described method. The rhizoplane cells were concentrated by centrifuging the PBS at 150 *g* for 15 min [24]. The pellet of cells was extracted for DNA as described before.

The DNA integrity was verified by agarose gel electrophoresis and its concentration was quantified using the Quant-iT PicoGreen dsDNA Assay Kit (Invitrogen, Waltham, MA) on a NanoDrop™ 3300 Fluorometer (Thermo Fisher Scientific, Waltham, MA). The primers 341F (5′-CCTACGGGNGGCWGCAG-3′) and 805R (5′-ACHVGGGTATCTAATCC-3′) were used to amplify the V3-V4 region of the 16S rRNA gene using the Mastercycler Nexus Gradient (Eppendorf, Germany) [25]. The PCR amplification steps included a denaturation step at 95 °C for 10 min followed by 25 cycles at 95 °C for 45 s, at 53 °C for 45 s, at 72 °C for 60 s, and a final step at 72 °C for 10 min. The PCR products were purified using the commercial FastGene™ columns (Nippon Genetics Co, Ltd) and quantified using the NanoDrop™ 3300 and PicoGreen. Quantification readings can be found in the Supplementary Table S1. Sequencing was done by Macrogen, Inc. (Seoul, South Korea) using the Illumina Miseq platform 2 × 300 bp.

## Bioinformatics Analyses: Taxonomic Annotation and Functional Prediction

The bioinformatics analyses of sequences were done using the “Quantitative insight into microbial ecology” v2020.8 (QIIME2) [26]. Denoising, dereplication, and chimera removal were done using the DADA2 [27]. This process resulted in a table of frequencies of amplicon sequence variants (ASVs) and a set of representative sequences of each ASV. Taxonomic annotation of ASVs was done with the representative sequences using the *classify-sklearn* plugin and a trained model with the Silva database v138 [28]. The Silva database training process was done with the *fit-classifier-naive-bayes* algorithm. All ASVs assigned to chloroplast and mitochondria were filtered out with the plugin *taxa filter-table* before statistical analyses. Putative metabolic functions were predicted using the “Phylogenetic investigation of communities by reconstruction of unobserved states” (PICRUSt v2.0) [29]. The abundance of metabolic routes was based on enzyme classification (EC) numbers, and the metabolic pathways were annotated with MetaCyc at levels 2 and 3 [30]. Bacterial taxonomic information was assigned into putative functional groups with the “Functional annotation of prokaryotic taxa” (FAPROTAX v1.2.6) software [31].

### Statistical Analysis

All statistical analyses were done in R [32]. Alpha diversity of bacterial taxa (ASVs) and putative metabolic pathways at level 3 of MetaCyc were determined using Hill numbers at different *q* orders (at *q* = 0, 1, and 2) [33]. Hill numbers were calculated using the hillR package (v0.5.1) [34]. Non-parametric analyses were used to determine the effect of plant part (rhizoplane, roots, and aerial parts) and time (1 and 5 months) on alpha diversity (Hill numbers at the order *q* = 0, 1, and 2). These analyses were conducted using the WRS2 package (v1.1–0) [35].

Beta diversity was investigated through ordination (principal component analysis (PCA)) and multivariate comparison (perMANOVA) using centered log-ratio transformed frequency tables (i.e., clr-transformation) of bacterial phyla and genera (taxonomic structure) and putative metabolic pathways at level 3 and putative functional groups (functional structure). The clr-transformation is necessary to analyze compositional high-throughput sequencing data [36]. The clr-transformation was done using the “aldex.clr” function within the ALDEx2 R package (v1.21.1) [36]. The effect of plant part (rhizoplane, roots, and aerial parts) and time (1 and 5 months) on bacterial taxonomic and functional structures were visualized with a PCA as given by the FactoMineR package (v2.3) [37], and their significance was determined with a permutational multivariate analysis of variance (perMANOVA; *n* = 999) with the vegan package (v2.5–7) [38]. Permutation multivariate analysis of dispersion (PERMDISP; *n* = 999) was computed using the “betadisper” function from the vegan package to determine differences in dispersion within treatments when the perMANOVA test indicated significant effects.

Taxonomic and functional beta diversity was partitioned into nestedness and turnover components to determine the extent of species replacement (turnover) or species loss (nestedness) explaining the Jaccard dissimilarity (beta diversity at order *q* = 0) between endophytic communities using the betapart R package (v1.5.6) [39]. Beta diversity of ASVs and putative metabolic pathways was partitioned for plants after one (roots and aerial parts) using the function “beta.pair” and after 5 months (rhizoplane, roots and aerial parts) using the “beta.multi” function of the betapart R package. Additionally, temporal partition (1 versus 5 months) was determined using the “beta.temp” function.

A compositional differential abundance analysis was used to determine the effect of plant part and time on bacterial phyla, genera, ASVs, putative metabolic pathways, and functional groups. ANOVA-like differential expression analysis (ALDEx) with Wilcoxon-test (function “aldex.ttest”) was used to compare taxa and functions between plant parts in one-month plants (roots vs aerial parts) and over time (1 vs 5 months), while a Kruskal–Wallis test (function “aldex.kw”) was used to compare plant parts (rhizoplane, roots, and aerial parts) in 5-month plants within ALDEx2 R package. Effect size, i.e., the difference between relative abundance of the bacterial groups divided by maximum dispersion within the plant parts and over time, was determined with R Package ALDEx2. The effect size was plotted versus the expected *p*-value for each feature in a volcano plot.

## Results

### Soil Characteristics

The loamy sand soil with a clay content of 45 ± 28 g kg^−1^, sand 784 ± 75 g kg^−1^, and loam 171 ± 67 g kg^−1^ had an average water content of 325 ± 107 g kg^−1^ and water holding capacity of 793 ± 201 g kg^−1^. The slightly acidic soil with a pH of 5.8 ± 0.4 and EC 0.35 ± 0.18 dS m^−1^ was rich in organic material with an organic C content of 65.5 ± 45.1 g kg^−1^, an inorganic C content of 0.79 ± 0.11 g kg^−1^, total N of 3.35 ± 1.99 g kg^−1^, and total P of 5.66 ± 2.50 mg kg^−1^.

### Bacterial Community Richness and Composition

A total of 487,609 high-quality filtered sequences were obtained from the roots (50 samples), aerial parts (53 samples), and rhizoplane (27 samples) of *A. religiosa*. Overall, 26 different bacterial phyla, 54 classes, 150 orders, 270 families, 486 genera, and 3887 ASVs were detected.

### The Bacterial Community and Putative Metabolic Functions in the Aerial Parts and Roots after 1 Month

**Fig. 1:**
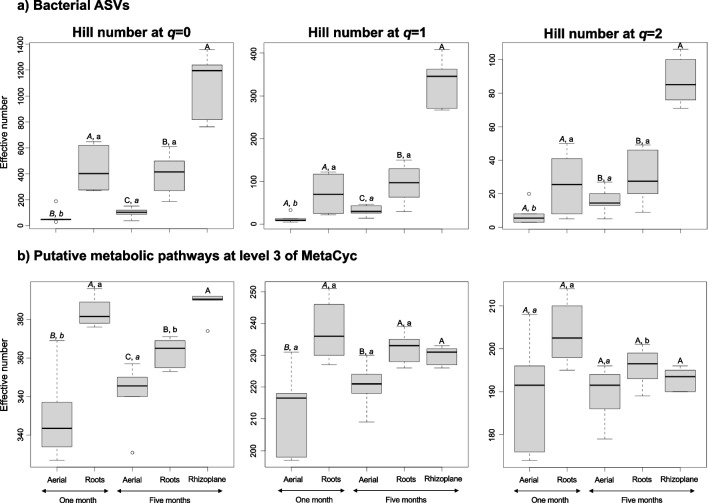
Boxplots with the Hill numbers based on **a** the bacterial amplicon sequence variants (ASVs) at *q* = 0, *q* = 1, and *q* = 2 and **b** the metabolic pathways at level 3 of MetaCyc as predicted with PICRUSt2 at *q* = 0, *q* = 1, and *q* = 2 in the aerial parts and roots after 1 month and aerial parts, rhizoplane, and roots of the conifer *Abies religiosa* (Kunth) Schltdl. & Cham. (oyamel) after 5 months. Different capital letters in italic (*A*, *B*) indicate a significant difference in Hill numbers between the aerial parts and the roots after one month, while different capital letters indicate a significant difference between the aerial parts, roots, and rhizoplane after 5 months (A, B, C) (*p* < 0.05). Different italic letters indicate a significant difference in Hill numbers between the aerial parts after 1 month compared to after 5 months (*a*, *b*) and different letters between the roots after 1 month compared to after 5 months (a, b)

After 1 month, the bacterial species richness (Hill number at *q* = 0), was significantly higher in the roots than in the aerial parts of *A. religiosa,* but not the number of frequent (Hill number at *q* = 1) and dominant ASVs (Hill number at *q* = 2) (*p* < 0.05; Fig. 1a).

**Fig. 2:**
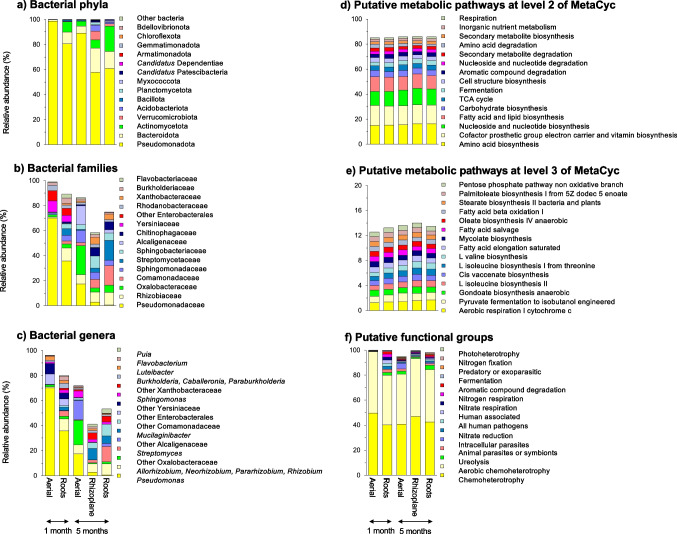
Barplots with the relative abundance (%) of 15 most abundant bacterial **a** phyla, **b** families, and **c** genera and **d** putative metabolic pathways at level 2 of MetaCyc and **e** level 3 and **f** putative functional groups as predicted with FAPROTAX in the aerial parts and roots after 1 month and aerial parts, rhizoplane, and roots of the conifer *Abies religiosa* (Kunth) Schltdl. & Cham. (oyamel) after 5 months

After 1 month, similar bacterial groups dominated in the aerial parts and roots of *A. religiosa* although their relative abundance was often different (Table S2; Fig. S2a, b). Nearly all bacteria in the aerial parts and roots belonged to the Pseudomonadota with Bacteroidota the second most abundant bacterial phyla in both plant parts (Fig. 2a). *Pseudomonas*, a member of the Pseudomonadaceae (Gammaproteobacteria), dominated in the aerial parts (71.6%) and the roots (37.9%), with members of *Luteibacter* (2.3%) the second most abundant bacterial genus in the aerial parts and *Rhizobium* in the roots (9.0%) (Fig. 2b, c).

**Fig. 3:**
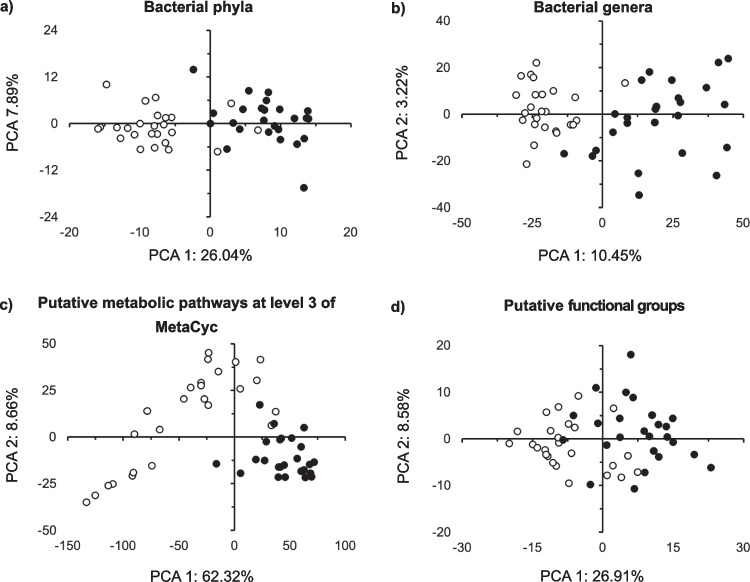
Principal component analysis (PCA) with all bacterial **a** phyla and **b** groups assigned up to the taxonomic level of genus, **c** putative metabolic pathways at level 3 of MetaCyc and **d** putative functional groups in the aerial parts of the conifer *Abies religiosa* (Kunth) Schltdl. & Cham. (oyamel) (◯) and in the roots (⬤) after 1 month

After 1 month, the bacterial community structure was highly significantly different between the roots and the aerial parts (*p* < 0.001) and the PCA separated both clearly (Table S3; Fig. 3a, b). Differences in the taxonomic structure at the phylum level between plant parts explained 17% of the variation and 8% at the genus level (Table S3). Loss of bacterial ASVs explained 71% of the differences in bacterial composition (Jaccard pairwise dissimilarity nestedness, 0.586; Table S4) between the roots and aerial parts, while the remaining 28% was explained by species replacement (turnover, 0.234).

The Hill numbers at *q* = 0 and *q* = 1 considering the putative metabolic pathways at level 3 of MetaCyc, as predicted with PICRUSt2, were significantly higher in the roots than in the aerial parts of *A. religiosa* after 1 month, but not at *q* = 2 (*p* < 0.05; Fig. 1b).

After 1 month, the most abundant putative metabolic pathway at level 2 was “Cofactor prosthetic group electron carrier and vitamin biosynthesis” (15.5%), while “Aerobic respiration I cytochrome c” (1.3%) at level 3 (Fig. 2d, e). Bacterial taxa were also assigned to putative functional groups with FAPROTAX, and “Chemoheterotrophy” and “Aerobic chemoheterotrophy” accounted for 87.4% of these groups (Fig. 2f). Significant differences were observed in most putative metabolic pathways and functional groups between the roots and the aerial parts after 1 month (*p* < 0.05; Fig. S2c, d, e). The PCA separated the functional structure of both plant parts based on metabolic pathways and functional groups, and the differences were significant (*p* < 0.001; Table S3 and Fig. 3c). The plant compartment explained 37% of the variation of the putative metabolic pathways. However, the analysis of the homogeneity of dispersion of the aerial parts and roots also indicated a significant difference when considering the putative metabolic pathways at level 3 (*p* = 0.017), as the functional structure varied more in the aerial parts (Fig. 3c). The Jaccard pairwise dissimilarity between the roots and aerial parts based on the putative metabolic pathways was low and explained solely by loss of metabolic pathways (Table S4).

### The Bacterial Community and Putative Metabolic Functions in the Different Plant Parts After 5 Months

After 5 months, Hill numbers at *q* = 0 and *q* = 1 based on the bacterial ASVs were significantly higher in the roots than in aerial parts, but significantly lower than in the rhizoplane (*p* < 0.05; Fig. 1a).

**Fig. 4:**
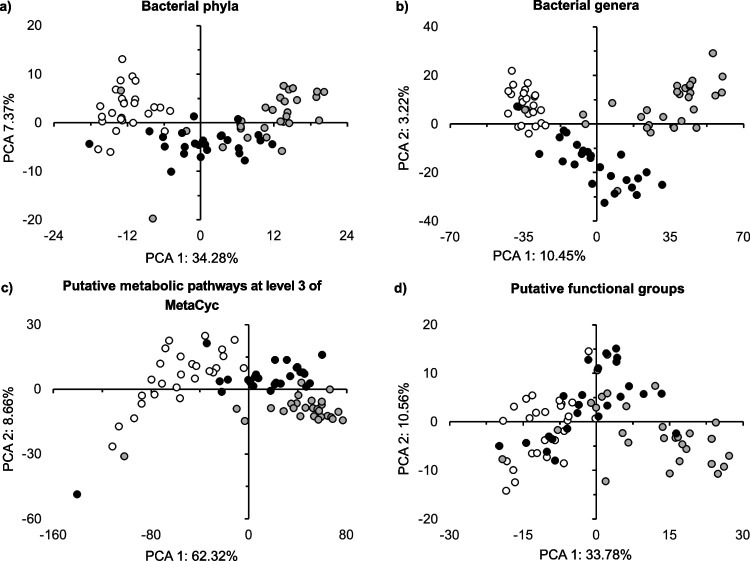
Principal component analysis (PCA) with all bacterial **a** phyla and **b** groups assigned up to the taxonomic level of genus, **c** putative metabolic pathways at level 3 of MetaCyc and **d** putative functional groups in the aerial parts of the conifer *Abies religiosa* (Kunth) Schltdl. & Cham. (oyamel) (◯), rhizoplane (  ), and in the roots (⬤) after 5 months

The relative abundance of the bacterial groups in the roots, aerial parts, and rhizoplane showed large significant differences (*p* < 0.05; Table S2; Fig. S3a). After 5 months, Pseudomonadota still dominated in the aerial parts, roots, and rhizoplane of *A. religiosa* (Fig. 2a)*.* The bacterial genus that was the most abundant, however, was different as *Mucilaginibacter* dominated in the rhizoplane (9.0%), *Streptomyces* in the roots (12.2%), and *Pseudomonas* in the aerial parts (18.1%) (Fig. 2c). Consequently, the bacterial community structure was highly significantly different between the different plant parts (*p* < 0.001) and the PCA separated both clearly (Table S3; Fig. 4a, b). The plant compartment explained 39% of the variation in the bacterial structure at the phylum level, 20% at the genus level, and 5% at the ASV level (Table S3). The dispersion analysis indicated a significant effect when the aerial plant parts were compared with the roots considering the bacterial genera (*p* = 0.019), being more dispersed in the roots. The Jaccard multiple-site dissimilarities between the rhizoplane, roots, and aerial parts were explained mostly by substitution and less by a loss of bacterial ASVs (Table S4).

After 5 months, Hill numbers at *q* = 0 based on the putative metabolic pathways at level 3 were significantly higher in the roots than in aerial parts, but significantly lower than in the rhizoplane (*p* < 0.05; Fig. 1).

After 5 months, “Amino acid biosynthesis” (16.1%) was the most abundant putative metabolic pathway at level 2, while “Aerobic respiration I cytochrome c” (1.6%) was at level 3 (Fig. 2c, d). “Chemoheterotrophy” and “Aerobic chemoheterotrophy” continued to dominate the putative functional groups (Fig. 2f). Most putative metabolic pathways and functional groups significantly differed between the rhizoplane or roots and the aerial parts (*p* < 0.05; Fig. S3b). The functional structures of the rhizoplane, roots, and aerial remained separated, and the differences between the plant parts were highly significant (*p* < 0.001; Table S3; Fig. 4c, d). The plant compartment explained 40% of the variation of the putative metabolic pathways. The Jaccard multiple-site dissimilarities between the rhizoplane, roots, and aerial parts considering the putative metabolic pathways at level 3 were explained mostly by loss of and less by replacement of metabolic pathways (Table S4).

### Changes Over Time in the Bacterial Community and Putative Metabolic Functions in Aerial Parts and Roots

The Hill numbers at *q* = 0, 1, and 2 were significantly higher in the aerial parts of *A. religiosa* after 5 months than after 1 month*,* but not in the roots (*p* < 0.05; Fig. 1a).

**Fig. 5:**
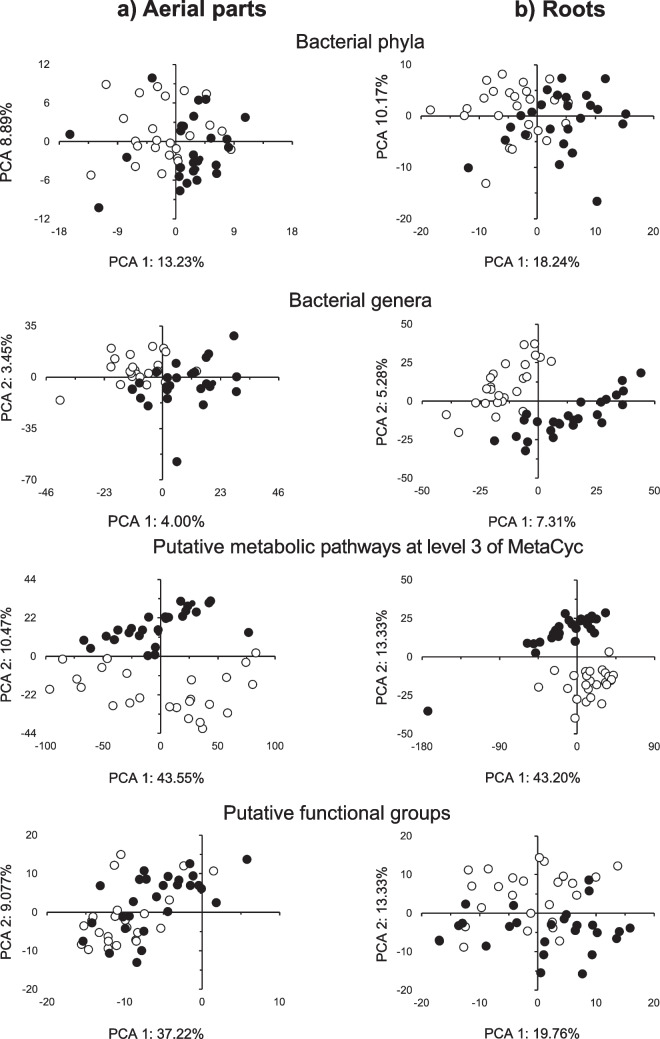
Principal component analysis (PCA) with all **a** bacterial phyla in the aerial parts and **b** roots, **c** bacterial groups assigned up to the taxonomic level of genus in the aerial parts and **d** roots, **e** putative metabolic pathways at level 3 of MetaCyc in the aerial parts and **f** roots, and **g** putative functional groups in the aerial parts and **h** the roots of the conifer *Abies religiosa* (Kunth) Schltdl. & Cham. (oyamel) after 1 month (◯) and 5 months (⬤)

The relative abundance of a small number of bacterial groups decreased or increased significantly in the aerial parts over time (i.e., 5 months versus 1 month) (*p* < 0.05; Fig. S4a). In the roots, however, many more bacterial genera showed large and significant changes over time (*p* < 0.05; Fig. S4b). The PCA separated the bacterial taxonomic structure in the aerial parts and roots after 5 months from that after 1 month, and the effect was significant (*p* < 0.05; Fig. 5a, b; Table S3). Time explained low taxonomic structure variation (< 6%) at the different levels. Dissimilarities in taxonomic composition between the roots and aerial parts after 1 month compared to after 5 months were explained nearly completely by the replacement of species (Table S4).

The Hill number at *q* = 0 based on putative metabolic pathways at level 3 was significantly higher in the aerial parts *A. religiosa* after 5 months than after 1 month but showed an opposite trend in the roots (*p* < 0.05; Fig. 1b).

Some putative metabolic pathways showed significant changes in the roots and aerial parts over time (*p* < 0.05; Table S2; Fig. S4c, d). For example, the relative abundance of “Chemoheterotrophy” and “Aerobic chemoheterotrophy” decreased significantly in both aerial parts and roots (*p* < 0.05; Table S2). The PCA separated the structure of the putative metabolic functions in the aerial parts and roots after 5 months from that after 1 month, and the effect was significant (*p* < 0.05; Fig. 5a, b; Table S3). The dissimilarities in functional composition between the roots and aerial parts after 1 month compared to after 5 months were low and explained nearly completely by the replacement of the putative metabolic pathways (Table S4).

## Discussion

After 1 month, many of the initial endophytic colonizers may originate from the seeds through vertical transmission, with fewer bacteria sourced from the environment. For instance, the roots may acquire them from the moss they penetrated, while the aerial parts may get them from wind-dispersed bacteria. Our observation of the limited degree of species replacement explaining the differences in community composition between the roots and aerial parts indicated that they likely originate from the same species pool, potentially belonging to Pseudomonadaceae [40] (Table S4). Additionally, we identified four bacterial genera unique to aerial parts and 162, albeit in very low abundance, in the roots, potentially representing recently acquired environmental bacteria. Another interesting finding is that at this early plant development stage, bacterial diversity tends to be lower in aerial parts compared to roots, a pattern seen in various plant species [e.g., 2, 22] (Fig. 1). The difference in bacterial diversity and structure between aerial parts and roots might relate to differences in environmental conditions. Conditions within seeds possibly resemble those in roots more closely than those in aerial parts. Our findings also indicate a higher abundance of putative aerobic chemoheterotrophy and respiration in aerial parts than in roots, suggesting oxidative conditions in the aerial parts that favor aerobic bacteria, while roots potentially select for anaerobic or facultative anaerobic bacteria (Table S5). Further investigations into the bacterial communities within the seeds and the moss could provide additional insights into these hypotheses.

After 5 months, the roots penetrated the mineral soil. As a result, the soil bacteria adhered to them, forming the rhizoplane (a bacterial community on the roots). This process led to the establishment of a bacterial rhizospheric community. Soil bacteria started colonizing the roots and eventually replaced some of the bacteria that were already present in the plant. This was evidenced by the partition of the beta diversity analysis (Table S4). We hypothesized that the bacterial diversity in the roots would be considerably higher after 5 months, but instead, the bacteria in the roots were predominantly replaced, maintaining the number of bacterial species within a limited range. We still found a high degree of nestedness between plant parts, confirming our second hypothesis, which suggests that the main source of endophytic bacteria was the soil bacteria, while in the aerial parts of the seedlings, the wind-dispersed bacteria may remain as a secondary source. The number of bacterial genera exclusive to the rhizoplane was almost ten times greater than those exclusively found in the roots. This indicated that the rhizoplane harbored a wide range of bacteria that did not colonize the roots or could be found in the aerial parts. The roots, however, also contained 24 bacterial genera that were not found in the aerial parts or the rhizoplane. This indicates that while some bacteria entered the roots, they did not migrate upwards to the shoots or originate from the rhizoplane. Interestingly, the diversity of metabolic pathways decreased in the roots over time, indicating that the plant selects functional traits over taxonomic groups with time.

### Shifts in Taxonomic Groups

González-Escobedo et al. [41] studied bacterial endophytes in roots, phloem and bark of healthy saplings of *Pinus arizonica* (Arizona pine) and *P. durangensis* (Durango pine) (< 3 m tall, 10 cm diameter) and found Enterobacteriaceae as the most abundant family in all samples, followed by Acetobacteraceae and Acidobacteriaceae. Although these bacterial families also dominated in needles of mature coniferous trees [42], the sum of their relative abundance in *A. religiosa* was only 2.3% in the roots and only 0.8% in the aerial parts after 5 months (Fig. 2b). In this study, members of the Pseudomonadaceae family dominated in the roots (35.8%) and aerial parts (70.1%) after 1 month, while Oxalobacteraceae (23.2%) dominated in the aerial parts and Streptomycetaceae (15.9%) in the roots after 5 months. Some bacterial families that dominated in young lodgepole pine as reported by Padda et al. [43] (i.e., Rhizobiaceae, Xanthobacteraceae, and Comamonadaceae) had also large relative abundances in *A. religiosa*. As such, the type and age of the plant, environmental conditions, and soil characteristics will determine which bacterial groups dominate the endophytic bacterial community.

In this study, *Pseudomonas* was the most abundant bacterial group in the aerial parts and roots after 1 month (Fig. 2c). Members of *Pseudomonas* are metabolically versatile and often the most dominant endophytes in leguminous and non-leguminous plants [44]. Some are beneficial for their hosts [45] and are capable of N_2_-fixation [46], but some are pathogens of plants such as *P. syringae* [47]. Their high relative abundance, especially in the aerial parts after 1 month, would suggest that they were dominant in the seeds or colonized early the young seedlings. Simonin et al. [40] in a meta-analysis of 63 seed microbiota studies covering 50 plant species found that five ASVs affiliated with *Pseudomonas* (e.g., *P. viridiflava* and *P. fluorescens*) were part of the core taxa. Which role *Pseudomonas* might play in the emerging *A. religiosa* and why it was so dominant remains to be investigated. Their N_2_ fixing capacity suggests that they might help the seedlings obtain nitrogen before the roots reach the mineral soil. This would explain the sharp drop in relative abundance of *Pseudomonas* when the roots had reached the mineral soil after 5 months. However, other characteristics of *Pseudomonas* might be important for the developing seedlings as endophytes are known to act against pathogens, produce plant growth-promoting molecules, and solubilize phosphorus, potassium, and other micro-nutrients [e.g., 48].

Although the relative abundance of *Pseudomonas* dropped sharply, it was still the dominant bacterial genus in the aerial parts of the conifer after 5 months, but in the roots, *Streptomyces* was the most abundant genus, and *Mucilaginibacter* in the rhizoplane (Fig. 2c). These changes might be due to changes in the plant environment, a selective process by the plant, or vertical transfer of some bacteria. *Streptomyces* has been reported as an endophyte of banana (*Musa* spp.) plantlets with plant growth-promoting capacities producing indole acetic acid and siderophores, solubilizing P, and with antagonistic activity against *Fusarium oxysporum* f. sp. *Cubense* [49]. It has been found in the root of plants, such as *Arabidopsis thaliana* [50] and wheat (*Triticum aestivum*) [51]. Some members of *Mucilaginibacter*, the most dominant bacterial genus in the rhizoplane*,* have been described as chitinolytic, Gram-negative, capsule-forming [52], and as root and/or shoot endophytes of fall dandelion (*Scorzoneroides autumnalis*) [53], grass “Kleine Fontane” (*Miscanthus sinensis*) [54], and Marama bean (*Tylosema esculentum*) [55]. They were also abundantly found in the roots of *A. religiosa* after 1 (2.7%) and 5 months (5.9%), but less so in the aerial parts after 1 (0.04%) and 5 months (0.5%). Other bacterial genera, e.g., *Rhizobium*, *Streptomyces*, and *Luteibacter*, that were abundant in the aerial parts and roots of *A. religiosa* have been reported as endophytes with the capacity to aid plants in their development (Table S5). They often combine plant growth-promoting capacities with antagonism against pathogens.

Of the 24 bacterial genera found only in the roots, the most abundant were *Paracoccus* and *Rubrobacter*. Nakkeeran et al. [49] reported that *Paracoccus* as endophytes promoting the growth of bananas*.* Members of *Rubrobacter* tolerate extreme conditions, such as drought and radiation stress (*R. radiotolerans*), some are salt tolerant [56], and the genus was found to be more abundant in *Fusarium* wilt-suppressive soils than in non-repressive ones [57]. Members of *Rubrobacter* have been described as endophytes in the roots of the orchid *Phalaenopsis* [58], and their tolerance to extreme conditions might help them survive as endophytes.

Four genera (i.e., *Frondihabitans, Staphylococcus*, *Lactococcus*, and *Fusobacterium*) were found only in the aerial parts, but not in the roots or rhizoplane. Nakkeeran et al. [49] reported that one of them (i.e., *Staphylococcus*) is an endophyte that promotes the growth of banana plantlets*.* Of the four genera found uniquely in the aerial parts, three genera were not found in the plant after one month (i.e., *Staphylococcus*, *Lactococcus*, and *Fusobacterium*), which raises the question of how they were acquired. It remains to be determined whether they originated from epiphytes, migrated from the rhizoplane through the plant, or were the product of specific selection (i.e., initially present in the rhizoplane and/or roots but later disappeared or undetected with the current method). A more comprehensive investigation utilizing shotgun metagenomics and increased sampling over time may be necessary to address this question.

### Shifts in Putative Metabolic Pathways and Functional Groups

Acuña et al. [59] reported that chemoheterotrophy and fermentation were the most abundant putative microbial functional groups within the endophytic bacterial community in seeds of the commercial vegetables Apiaceae (parsley and carrot), Asteraceae (lettuce), Brassicaceae (cabbage and broccoli), and Solanaceae (tomato). In *A. religiosa,* chemoheterotrophy and aerobic chemoheterotrophy dominated in both the aerial parts and roots after 1 and 5 months, but fermentation was less abundant. It is possible that chemoheterotrophs, i.e., microorganisms dependent on external C molecules rather than synthesizing their organic molecules (https://bio.libretexts.org; Table S5), could potentially be vertically transmitted and be the initial plant colonizers as they can use carbohydrates produced by the plant. As emerging pine tree plants face complex physiological trade-offs in resource allocation, including chemical defense mechanisms [60], while chemoheterotrophy and aerobic chemoheterotrophy remained dominant in the seedlings, their relative abundance decreased over time. This change might indicate that the plant increased resource allocation towards defense mechanisms after 5 months. Consequently, these non-pathogenic yet opportunistic bacteria decreased in parallel with the overall bacterial diversity. A brief description of some of the putative metabolic pathways that differed between the plant parts, and over time is given in Table S6.

The differences in relative abundances of the putative metabolic functions in the different plant parts and their changes over time were much smaller than the variations of the bacterial groups (Fig. 2). It is well known that different bacteria might participate in a specific metabolic process. As such, the bacteria that participate in the specific metabolic process might change but not the metabolic process (i.e., the relative abundance of the bacterial groups that participate in the process might be more stochastic than the metabolic process and defined by current and previous environmental conditions). It should be remembered, however, that the detected changes in metabolic functions are putative. Other advanced techniques as reviewed by Dudeja et al. [61], such as metaproteomics, metatranscriptomics, metaproteogenomic, microRNAs, and microarrays, might be required to determine which endophytic bacteria participate in a certain process and how they might help in the development of the plantlets.

It is important to note that the metabolic functions described in this study were predicted from taxonomic data, which can sometimes underestimate gene frequencies [62] or perform weakly with environmental microbiomes [63]. While this analysis provides a broad understanding of putative metabolic pathways and functions, additional investigations into bacterial gene expression will be necessary to validate and further explore the role of plant endophytes in different plant parts over time. These roles might be related to antagonisms against pathogens and production of plant growth-promoting metabolites or providing the plants with essential nutrients.

## Conclusions

This study contributes to our understanding of bacterial endophytes in developing pine trees. Using metabarcoding of the 16S rRNA, we found that the bacterial community structure showed clear differences between the aerial parts and the roots after 1 month that became more accentuated after 5 months. The most dominant bacterial genera in roots and aerial parts of *A. religiosa* had plant growth-promoting capacities and/or were antagonistic against plant pathogens. Our findings showed that endophytes play an important role in emerging plants although their contribution to plant development might change over time.

### Supplementary Information

Below is the link to the electronic supplementary material.

Supplementary file1 (PDF 2529 KB)

Supplementary file2 (PDF 373 KB)

Supplementary file3 (PDF 616 KB)

Supplementary file4 (PDF 479 KB)

Supplementary file5 (DOCX 31 KB)

Supplementary file6 (DOCX 27 KB)

Supplementary file7 (DOCX 28 KB)

Supplementary file8 (DOCX 22 KB)

Supplementary file9 (DOCX 41 KB)

Supplementary file10 (DOCX 44 KB)

## Data Availability

All data analyzed are provided in the manuscript and supplemental files. The raw datasets generated and analyzed during the study are available from the corresponding author upon reasonable request. Raw sequence data can be found under the bioproject accession number PRJNA1024058. https://www.ncbi.nlm.nih.gov/bioproject/PRJNA1024058.
