# Supplementary material for: Spatial and Temporal Shifts of Endophytic Bacteria in Conifer Seedlings of Abies religiosa (Kunth) Schltdl. & Cham
Source: Microb Ecol. 2024 Jul 3;87:90. doi: 10.1007/s00248-024-02398-9 (PMC11222277; doi:10.1007/s00248-024-02398-9)

Plants were collected from two locations from three areas (4 m^2)
after one and five months

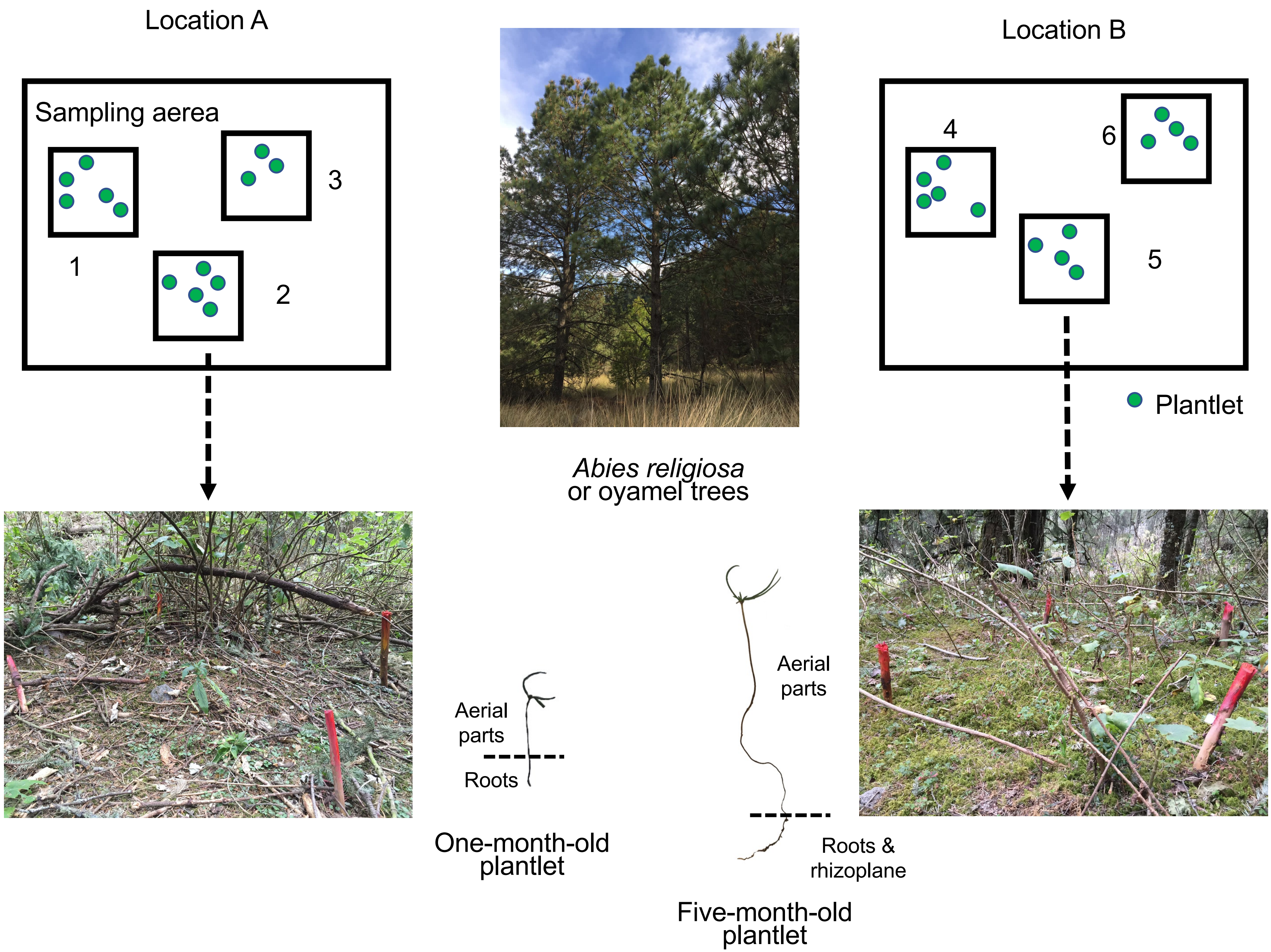

Supplement: Supplementary file 1 [file 248_2024_2398_MOESM1_ESM.pdf]
