# Supplementary material for: Spatial and Temporal Shifts of Endophytic Bacteria in Conifer Seedlings of Abies religiosa (Kunth) Schltdl. & Cham
Source: Microb Ecol. 2024 Jul 3;87:90. doi: 10.1007/s00248-024-02398-9 (PMC11222277; doi:10.1007/s00248-024-02398-9)
Supplement: Supplementary file 2 [file 248_2024_2398_MOESM2_ESM.pdf]

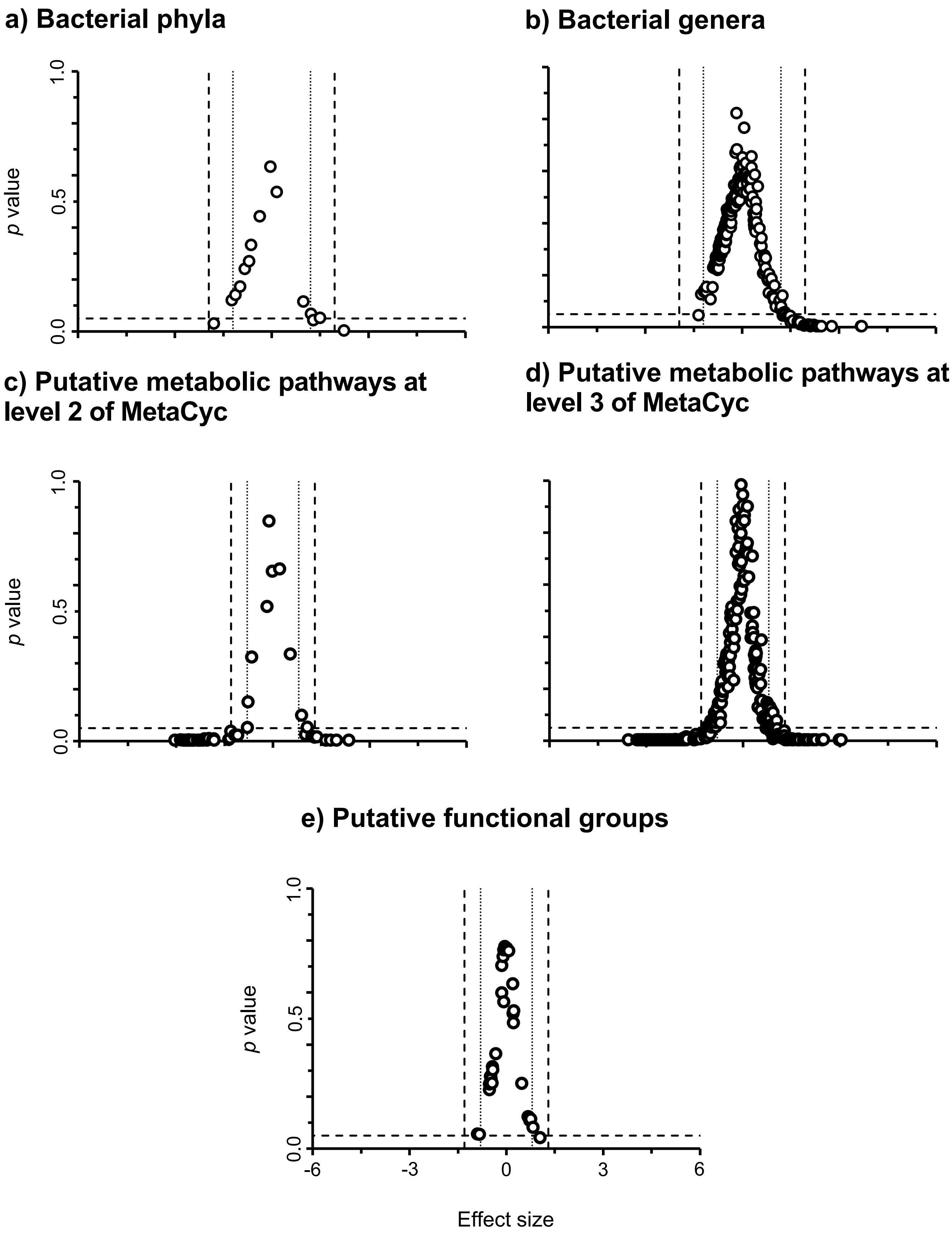

a) Bacterial phyla
b) Bacterial genera
c) Putative metabolic pathways at level 2 of MetaCyc
d) Putative metabolic pathways at level 3 of MetaCyc
e) Putative functional groups
p value
0.0
0.5
1.0
-6
-3
0
3
6
Effect size
