# Supplementary material for: Spatial and Temporal Shifts of Endophytic Bacteria in Conifer Seedlings of Abies religiosa (Kunth) Schltdl. & Cham
Source: Microb Ecol. 2024 Jul 3;87:90. doi: 10.1007/s00248-024-02398-9 (PMC11222277; doi:10.1007/s00248-024-02398-9)
Supplement: Supplementary file 5 [file 248_2024_2398_MOESM10_ESM.docx]

**Table S6** Some of the most affected putative metabolic functions as determined with PICRUST V2 level 2 [8] and FaproTax [14]

| ⎯⎯⎯⎯⎯⎯⎯⎯⎯⎯⎯⎯⎯⎯⎯⎯⎯⎯⎯⎯⎯⎯⎯⎯⎯⎯⎯⎯⎯⎯⎯⎯⎯⎯⎯⎯⎯⎯⎯⎯⎯⎯⎯⎯⎯⎯⎯⎯⎯⎯⎯⎯⎯⎯⎯⎯⎯⎯⎯⎯⎯⎯⎯⎯⎯⎯⎯⎯⎯ | | |
| --- | --- | --- |
| PICRUST V2 (MetaCyc) | Description | References and links |
| ⎯⎯⎯⎯⎯⎯⎯⎯⎯⎯⎯⎯⎯⎯⎯⎯⎯⎯⎯⎯⎯⎯⎯⎯⎯⎯⎯⎯⎯⎯⎯⎯⎯⎯⎯⎯⎯⎯⎯⎯⎯⎯⎯⎯⎯⎯⎯⎯⎯⎯⎯⎯⎯⎯⎯⎯⎯⎯⎯⎯⎯⎯⎯⎯⎯⎯⎯⎯⎯ | | |
| 1,5-anhydrofructose degradation | [1,5-anhydro-D-fructose](https://biocyc.org/compound?orgid=META&id=15-ANHYDRO-D-FRUCTOSE) is a monosaccharide that is formed directly from starch or glycogen through the activity of [EC 4.2.2.13, exo-(1→4)-α-D-glucan lyase](https://biocyc.org/META/NEW-IMAGE?type=EC-NUMBER&object=EC-4.2.2.13). (Bacteria)  Yu and Fiskesund [21] suggested that the pathway operates only when the organism is subjected to biotic and abiotic stresses | [5] <https://biocyc.org/META/NEW-IMAGE?object=PWY-6992&redirect=T>, [21] |
| Adenosylcobalamin biosynthesis II (aerobic) | [Adenosylcobalamin](https://biocyc.org/compound?orgid=META&id=ADENOSYLCOBALAMIN) also known as vitamin B12 or coenzyme B12  The best characterized aerobic [adenosylcobalamin](https://biocyc.org/compound?orgid=META&id=ADENOSYLCOBALAMIN) biosynthetic pathway is that of the aerobic bacterium [*Pseudomonas denitrificans*](https://biocyc.org/META/NEW-IMAGE?object=TAX-43306) | [5] https://biocyc.org/META/NEW-IMAGE?object=P381-PWY&redirect=T |
| Aerobic respiration I cytochrome c | Respiration is a process by which electrons are passed from an electron donor to a terminal electron acceptor and cytochrome c can be a member of the electron transfer chain (Eukaryotes, Bacteria (e.g., [Pseudomonas putida KT2440](https://biocyc.org/META/NEW-IMAGE?object=TAX-160488))) | [5] https://biocyc.org/META/NEW-IMAGE?type=PATHWAY&object=PWY-3781 |
|  |  |  |
| Alcohol degradation | Contains pathways of degradation of various monohydric, dihydric, and trihydric alcohols, yielding a source of nutrients and energy. | [5] https://biocyc.org/META/NEW-IMAGE?type=PATHWAY&object=Alcohol-Degradation |
| Aldehyde degradation |  | [5] https://biocyc.org/META/substring-search?type=NIL&object=Aldehyde+degradation |
| Amine and polyamine biosynthesis | Amines and polyamines play a variety of roles in metabolism, including acting as osmoprotectants, keeping DNA in a condensed state, and serving as intermediates in the synthesis of macromolecules. | [5] https://biocyc.org/META/NEW-IMAGE?type=PATHWAY&object=Polyamine-Biosynthesis |
| Amino acid biosynthesis | This class contains pathways for the biosynthesis of amino acids, including the 22 amino acids present normally in proteins, as well as the biosynthesis of other amino acids and the modification of amino acids incorporated into proteins. | [5] <https://biocyc.org/META/NEW-IMAGE?type=PATHWAY&object=Amino-Acid-Biosynthesis> |
| Amino acid degradation |  | [5] <https://biocyc.org/META/substring-search?type=NIL&object=Amino+acid+degradation> |
| Aminoacyl tRNA Charging | Contains the set of reactions by which the various amino acids become bonded to their corresponding tRNAs; the products of these reactions recognize cognate codons in mRNAs present in ribosomes and react there, forming peptide bonds, thereby, lengthening a growing polypeptide. | [5] https://biocyc.org/META/NEW-IMAGE?object=Aminoacyl-tRNAs-Charging&redirect=T |
| Aromatic compound degradation | contains pathways of degradation of various aromatic compounds, including heterocyclic compounds and those that contain sulfur. The pathways provide a source of nutrients and energy. | [5] https://biocyc.org/META/NEW-IMAGE?type=PATHWAY&object=AROMATIC-COMPOUNDS-DEGRADATION |
| Carbohydrate Biosynthesis | All carbohydrates follow the general formula: C_n_(H_2_O)_n_. Carbohydrates have a variety of different functions, which include acting as metabolic intermediates and storage forms of carbon and energy. Other carbohydrates constitute structural materials, while still others are indicators and antigens used for cell recognition. | https://bio.libretexts.org/Courses/University_of_California_Davis/BIS_105%3A__Biomolecules_and_Metabolism_(Murphy)/Carbohydrates  [5] <https://biocyc.org/META/NEW-IMAGE?type=PATHWAY&object=Carbohydrates-Biosynthesis> |
| ⎯⎯⎯⎯⎯⎯⎯⎯⎯⎯⎯⎯⎯⎯⎯⎯⎯⎯⎯⎯⎯⎯⎯⎯⎯⎯⎯⎯⎯⎯⎯⎯⎯⎯⎯⎯⎯⎯⎯⎯⎯⎯⎯⎯⎯⎯⎯⎯⎯⎯⎯⎯⎯⎯⎯⎯⎯⎯⎯⎯⎯⎯⎯⎯⎯⎯⎯⎯⎯ | | |

| **Table S6** Continued  ⎯⎯⎯⎯⎯⎯⎯⎯⎯⎯⎯⎯⎯⎯⎯⎯⎯⎯⎯⎯⎯⎯⎯⎯⎯⎯⎯⎯⎯⎯⎯⎯⎯⎯⎯⎯⎯⎯⎯⎯⎯⎯⎯⎯⎯⎯⎯⎯⎯⎯⎯⎯⎯⎯⎯⎯⎯⎯⎯⎯⎯⎯⎯⎯⎯⎯⎯⎯⎯ | | |
| --- | --- | --- |
| Carbohydrate degradation |  | [5] <https://biocyc.org/META/NEW-IMAGE?type=PATHWAY&object=Carbohydrates-Degradation> |
| Carboxylate degradation | This class contains pathways of utilization of aliphatic carboxylates to be utilized as sources of nutrients and energy. | [5] https://biocyc.org/META/NEW-IMAGE?type=PATHWAY&object=CARBOXYLATES-DEG |
| Cell Structure Biosynthesis |  | [5] https://biocyc.org/META/NEW-IMAGE?object=Cell-Structure-Biosynthesis&redirect=T |
| Chlorinated compound degradation | Under aerobic conditions chlorinated pollutants are difficult for microorganisms to degradable, but anaerobic bacteria can often metabolize them through organohalide respiration reactions. | [5] <https://biocyc.org/META/NEW-IMAGE?object=CHLORINATED-COMPOUNDS-DEG&redirect=T>, [16] |
| Cofactor prosthetic group electron carrier and vitamin biosynthesis | Contains pathways of biosynthesis of small molecules, including cofactors, prosthetic groups, electron carriers and vitamins, that participate in enzyme reactions. | [5] https://biocyc.org/META/NEW-IMAGE?type=ECOCYC-CLASS&object=Cofactor-Biosynthesis |
| Fatty acid and lipid degradation | contains pathways by which various fatty acids and other lipids, including phospholipids, are degraded to serve and sources of nutrients and energy. | [5] https://biocyc.org/META/NEW-IMAGE?object=Fatty-Acid-and-Lipid-Degradation&redirect=T |
| Glycan biosynthesis | Bieberich [3] defined N-glycans as oligosaccharides by their chemical nature, (i.e., branched chains of sugar residues attached to each other by α- and β-glycosidic linkages) | [5] https://biocyc.org/META/substring-search?type=NIL&object=Glycan+biosynthesis |
| Glyoxylate cycle | The glyoxylate cycle is a sequence of reactions that form metabolic intermediates for biosynthesis (anaplerotic reactions) that enables an organism to use substrates that enter central carbon metabolism at the level of [acetyl-CoA](https://biocyc.org/compound?orgid=META&id=ACETYL-COA) as the sole carbon source. Such substrates include fatty acids, alcohols, and esters (often the products of fermentation), as well as waxes, alkenes, and methylated compounds. The pathway does not occur in vertebrates, but it is found in plants and certain bacteria, fungi, and invertebrates. | [5] https://biocyc.org/META/NEW-IMAGE?type=PATHWAY&object=GLYOXYLATE-BYPASS |
| Methylaspartate cycle | Some organisms utilize organic substrates (such as fatty acids, alcohols, esters, as well as waxes, alkenes, and methylated compounds) that are metabolized via [acetyl-CoA](https://biocyc.org/compound?orgid=META&id=ACETYL-COA). These organisms need to find a way to convert [acetyl-CoA](https://biocyc.org/compound?orgid=META&id=ACETYL-COA), a 2-carbon compound, to a 4-carbon compound that could feed reactions that form metabolic intermediates for biosynthesis (Halobacteria) | [5] https://biocyc.org/META/NEW-IMAGE?object=PWY-6728&redirect=T |
| Other degradation utilization | Contains pathways of degradation of substrates with chemical compositions that do not fit into any of the other classes within the degradation/utilization/assimilation group | [5] https://biocyc.org/META/NEW-IMAGE?object=Other-Degradation&redirect=T |
| Polymeric compound degradation | Includes pathways for the degradation of complex polymers | [5] https://biocyc.org/META/NEW-IMAGE?object=Polymer-Degradation&redirect=T |
| ⎯⎯⎯⎯⎯⎯⎯⎯⎯⎯⎯⎯⎯⎯⎯⎯⎯⎯⎯⎯⎯⎯⎯⎯⎯⎯⎯⎯⎯⎯⎯⎯⎯⎯⎯⎯⎯⎯⎯⎯⎯⎯⎯⎯⎯⎯⎯⎯⎯⎯⎯⎯⎯⎯⎯⎯⎯⎯⎯⎯⎯⎯⎯⎯⎯⎯⎯⎯⎯ | | |

**Table S6** Continued

| ⎯⎯⎯⎯⎯⎯⎯⎯⎯⎯⎯⎯⎯⎯⎯⎯⎯⎯⎯⎯⎯⎯⎯⎯⎯⎯⎯⎯⎯⎯⎯⎯⎯⎯⎯⎯⎯⎯⎯⎯⎯⎯⎯⎯⎯⎯⎯⎯⎯⎯⎯⎯⎯⎯⎯⎯⎯⎯⎯⎯⎯⎯⎯⎯⎯⎯⎯⎯⎯ | | |
| --- | --- | --- |
| Respiration | Respiration is a process by which electrons are passed from an electron donor to a terminal electron acceptor | [5] https://biocyc.org/META/NEW-IMAGE?type=PATHWAY&object=PWY-3781 |
| Superpathway of chorismate metabolism | [chorismate](https://biocyc.org/compound?orgid=META&id=CHORISMATE) is the principal common precursor of the aromatic amino acids [L-tryptophan](https://biocyc.org/compound?orgid=META&id=TRP), [L-tyrosine](https://biocyc.org/compound?orgid=META&id=TYR) and [L-phenylalanine](https://biocyc.org/compound?orgid=META&id=PHE), as well as the essential compounds [5,6,7,8-tetrahydrofolate](https://biocyc.org/compound?orgid=META&id=THF), [ubiquinone-*8*](https://biocyc.org/compound?orgid=META&id=UBIQUINONE-8), [menaquinol-*8*](https://biocyc.org/compound?orgid=META&id=REDUCED-MENAQUINONE) and [enterobactin](https://biocyc.org/compound?orgid=META&id=ENTEROBACTIN) (enterochelin) [17] | [5] https://biocyc.org/META/NEW-IMAGE?type=PATHWAY&object=ALL-CHORISMATE-PWY&show-citations=NIL |
| Superpathway of fucose and rhamnose degradation | In [*Escherichia coli*](https://biocyc.org/META/NEW-IMAGE?object=TAX-562), [L-fucose](https://biocyc.org/META/NEW-IMAGE?type=COMPOUND&object=L-fucoses) and [L-rhamnose](https://biocyc.org/META/NEW-IMAGE?type=COMPOUND&object=L-rhamnose) are metabolized through parallel pathways. The pathways converge after their corresponding aldolase reactions yielding the same products: [glycerone phosphate](https://biocyc.org/compound?orgid=META&id=DIHYDROXY-ACETONE-PHOSPHATE) (which enters central metabolism directly via the glycolysis pathway) and [(*S*)-lactaldehyde](https://biocyc.org/compound?orgid=META&id=LACTALD). Under aerobic conditions, [(*S*)-lactaldehyde](https://biocyc.org/compound?orgid=META&id=LACTALD) is oxidized in two steps to [pyruvate](https://biocyc.org/compound?orgid=META&id=PYRUVATE), thereby channeling all the carbons from fucose or rhamnose into central metabolic pathways. Under anaerobic conditions, [(*S*)-lactaldehyde](https://biocyc.org/compound?orgid=META&id=LACTALD) is reduced to [(*S*)-propane-1,2-diol](https://biocyc.org/compound?orgid=META&id=PROPANE-1-2-DIOL), which is secreted into the environment. | [5] https://biocyc.org/META/NEW-IMAGE?object=FUC-RHAMCAT-PWY&redirect=T |
| Superpathway of Glycolysis pyruvate dehydrogenase TCA and glyoxylate bypass | Integrates some of the fundumental components of energy metabolism, starting with a hexose sugar and ending with [CO_2_](https://biocyc.org/compound?orgid=META&id=CARBON-DIOXIDE) and several forms of highly reducing metabolites that can be used for ATP generation. | [5] https://biocyc.org/META/NEW-IMAGE?object=GLYCOLYSIS-TCA-GLYOX-BYPASS&redirect=T |
| TCA cycle | The tricarboxylic acid (TCA) cycle or the Krebs cycle is a series of [biochemical reactions](https://en.wikipedia.org/wiki/Chemical_reaction) to release the energy stored in [nutrients](https://en.wikipedia.org/wiki/Nutrient) through the [oxidation](https://en.wikipedia.org/wiki/Redox) of [acetyl-CoA](https://en.wikipedia.org/wiki/Acetyl-CoA) derived from [carbohydrates](https://en.wikipedia.org/wiki/Carbohydrate), [fats](https://en.wikipedia.org/wiki/Fat), and [proteins](https://en.wikipedia.org/wiki/Protein). The chemical energy released is available under the form of [ATP](https://en.wikipedia.org/wiki/Adenosine_triphosphate). The [Krebs](https://en.wikipedia.org/wiki/Hans_Krebs_(biochemist)) cycle is used by [organisms](https://en.wikipedia.org/wiki/Organism) that [respire](https://en.wikipedia.org/wiki/Cellular_respiration) (as opposed to organisms that [ferment](https://en.wikipedia.org/wiki/Fermentation)) to generate energy, either by [anaerobic respiration](https://en.wikipedia.org/wiki/Anaerobic_respiration) or [aerobic respiration](https://en.wikipedia.org/wiki/Aerobic_respiration). | <https://en.wikipedia.org/wiki/Citric_acid_cycle>  [5] https://biocyc.org/META/substring-search?type=NIL&object=TCA+cycle |
| ⎯⎯⎯⎯⎯⎯⎯⎯⎯⎯⎯⎯⎯⎯⎯⎯⎯⎯⎯⎯⎯⎯⎯⎯⎯⎯⎯⎯⎯⎯⎯⎯⎯⎯⎯⎯⎯⎯⎯⎯⎯⎯⎯⎯⎯⎯⎯⎯⎯⎯⎯⎯⎯⎯⎯⎯⎯⎯⎯⎯⎯⎯⎯⎯⎯⎯⎯⎯⎯ | | |
| FaproTax |  |  |
| ⎯⎯⎯⎯⎯⎯⎯⎯⎯⎯⎯⎯⎯⎯⎯⎯⎯⎯⎯⎯⎯⎯⎯⎯⎯⎯⎯⎯⎯⎯⎯⎯⎯⎯⎯⎯⎯⎯⎯⎯⎯⎯⎯⎯⎯⎯⎯⎯⎯⎯⎯⎯⎯⎯⎯⎯⎯⎯⎯⎯⎯⎯⎯⎯⎯⎯⎯⎯⎯ | | |
| Aerobic chemoheterotrophy | Chemoheterotrophs cannot synthesize their own organic molecules. They ingest earlier formed carbon molecules, such as carbohydrates and lipids, synthesized by other organisms. However, like chemoautotrophs they can still obtain energy from the oxidation of inorganic molecules (e.g., [4]) |  |
| Aliphatic non methane hydrocarbon degradation | Bacteria can degrade aliphatic saturated and unsaturated hydrocarbons via aerobic and anaerobic pathways [1] |  |
| Anoxygenic photoautotrophy | Unlike oxygenic phototrophic bacteria such as algae and cyanobacteria, anoxygenic phototrophic can use both organic and inorganic electron donors for light-dependent fixation of carbon dioxide without generating oxygen. | [9] |
| ⎯⎯⎯⎯⎯⎯⎯⎯⎯⎯⎯⎯⎯⎯⎯⎯⎯⎯⎯⎯⎯⎯⎯⎯⎯⎯⎯⎯⎯⎯⎯⎯⎯⎯⎯⎯⎯⎯⎯⎯⎯⎯⎯⎯⎯⎯⎯⎯⎯⎯⎯⎯⎯⎯⎯⎯⎯⎯⎯⎯⎯⎯⎯⎯⎯⎯⎯⎯⎯ | | |

**Table S6** Continued

| ⎯⎯⎯⎯⎯⎯⎯⎯⎯⎯⎯⎯⎯⎯⎯⎯⎯⎯⎯⎯⎯⎯⎯⎯⎯⎯⎯⎯⎯⎯⎯⎯⎯⎯⎯⎯⎯⎯⎯⎯⎯⎯⎯⎯⎯⎯⎯⎯⎯⎯⎯⎯⎯⎯⎯⎯⎯⎯⎯⎯⎯⎯⎯⎯⎯⎯⎯⎯⎯ | | |
| --- | --- | --- |
| Anoxygenic photosynthesis | Photosynthetic CO_2_ fixation by cyanobacteria (oxygenic phototrophs), and purple and green sulfur bacteria (anoxygenic phototrophs) [10]  A special form of [photosynthesis](https://en.wikipedia.org/wiki/Photosynthesis) used by some [bacteria](https://en.wikipedia.org/wiki/Bacteria) and [archaea](https://en.wikipedia.org/wiki/Archaea), which differs from the better known [oxygenic photosynthesis](https://en.wikipedia.org/wiki/Photosynthesis) in algae, cyanobacteria and [plants](https://en.wikipedia.org/wiki/Plant) in the [reductant](https://en.wikipedia.org/wiki/Reductant) used (e.g. [hydrogen sulfide](https://en.wikipedia.org/wiki/Hydrogen_sulfide)) and the [byproduct](https://en.wikipedia.org/wiki/Byproduct) generated (e.g. elemental [sulfur](https://en.wikipedia.org/wiki/Sulfur)) is different from that used in [oxygenic photosynthesis](https://en.wikipedia.org/wiki/Photosynthesis) in [plants](https://en.wikipedia.org/wiki/Plant) ([water](https://en.wikipedia.org/wiki/Water)) generated ([molecular oxygen](https://en.wikipedia.org/wiki/Dioxygen_in_biological_reactions)). | https://en.wikipedia.org/wiki/Anoxygenic_photosynthesis |
| Aromatic compound degradation | contains pathways of degradation of various aromatic compounds, including heterocyclic compounds and those that contain sulfur. The pathways provide a source of nutrients and energy. | [5] https://biocyc.org/META/NEW-IMAGE?type=PATHWAY&object=AROMATIC-COMPOUNDS-DEGRADATION |
| Aromatic compound degradation | contains pathways of degradation of various aromatic compounds, including heterocyclic compounds and those that contain sulfur. The pathways provide a source of nutrients and energy. | [5] https://biocyc.org/META/NEW-IMAGE?type=PATHWAY&object=AROMATIC-COMPOUNDS-DEGRADATION |
| Cellulolysis | Cellulolysis is essentially the hydrolysis of cellulose | https://www.e-education.psu.edu/egee439/node/669 |
| Chemoheterotrophy | Chemoheterotrophs cannot synthesize their own organic molecules. They ingest earlier formed carbon molecules, such as carbohydrates and lipids, synthesized by other organisms. However, like chemoautotrophs they can still obtain energy from the oxidation of inorganic molecules.  Chemoheterotrophs are the most abundant type of chemotrophic organisms | https://bio.libretexts.org/Bookshelves/Microbiology/Microbiology_(Boundless)/05%3A_Microbial_Metabolism/5.01%3A_Types_of_Metabolism/5.1B%3A_Chemoautotrophs_and_Chemohetrotrophs |
| Dark hydrogen oxidation | Anantharaman [2] described uncultured sulfur oxidizing Gammaproteobacteria (SUP05 group) from the deep sea. They contain and express genes encoding group 1 Ni, Fe hydrogenase enzymes for H_2_ oxidation and are found in different marine environments |  |
| Methanol oxidation | Methanol is used as their sole sources of carbon and energy [7] | [12] |
| Methylotrophy | Methylotrophic bacteria use reduced carbon substrates containing no carbon-carbon bonds (C1) (e.g., methanol, methane and other methylated compounds) as their sole sources of carbon and energy [7] |  |
| Nitrate, nitrite and nitrous oxide denitrification | The reduction of nitrate to nitrite, nitrite to nitric oxide, nitric oxide to nitrous oxide and dinitrogen | e.g. [15] |
| Nitrate reduction | Assimilatory and dissimilatory nitrate reduction to ammonium, and denitrification reduction of nitrate to nitrous oxide or dinitrogen | [6, 18] |
| Nitrate respiration | Nitrate is the starting point in assimilatory and respiratory nitrogen pathways [13] |  |
| Nitrite denitrification | The reduction of nitrite to nitric oxide and further to nitrous oxide and denitrogen |  |
| Nitrogen fixation | A process by which nitrogen gas is converted into biologically available ammonia | e.g. [19, 20] |
| Nitrogen reduction |  |  |
| Nitrogen respiration |  |  |
| ⎯⎯⎯⎯⎯⎯⎯⎯⎯⎯⎯⎯⎯⎯⎯⎯⎯⎯⎯⎯⎯⎯⎯⎯⎯⎯⎯⎯⎯⎯⎯⎯⎯⎯⎯⎯⎯⎯⎯⎯⎯⎯⎯⎯⎯⎯⎯⎯⎯⎯⎯⎯⎯⎯⎯⎯⎯⎯⎯⎯⎯⎯⎯⎯⎯⎯⎯⎯⎯ | | |

**Table S6** Continued

| ⎯⎯⎯⎯⎯⎯⎯⎯⎯⎯⎯⎯⎯⎯⎯⎯⎯⎯⎯⎯⎯⎯⎯⎯⎯⎯⎯⎯⎯⎯⎯⎯⎯⎯⎯⎯⎯⎯⎯⎯⎯⎯⎯⎯⎯⎯⎯⎯⎯⎯⎯⎯⎯⎯⎯⎯⎯⎯⎯⎯⎯⎯⎯⎯⎯⎯⎯⎯⎯ | | |
| --- | --- | --- |
| Photoautotrophy | Converting of sunlight into ATP and use it to fix carbon dioxide into organic compounds (the Calvin cycle)  Photoautotrophy in the green sulfur bacterium Prosthecochloris aestaurii can be driven by either electrons from a solid electrode or acetate oxidation via direct interspecies electron transfer from a heterotrophic partner bacterium, Geobacter sulfurreducens [10] | https://bio.libretexts.org/Bookshelves/Microbiology/Microbiology_(Bruslind)/15%3A_Phototrophy |
| Photoheterotrophy | Converting of sunlight into ATP but utilize pre-made organic compounds available in the environment. | https://bio.libretexts.org/Bookshelves/Microbiology/Microbiology_(Bruslind)/15%3A_Phototrophy |
| Phototrophy | “Light eating” refers to the process by which energy from the sun is captured and converted into chemical energy in the form of ATP.  Photosynthetic CO_2_ fixation by cyanobacteria (oxygenic phototrophs), and purple and green sulfur bacteria (anoxygenic phototrophs) | https://bio.libretexts.org/Bookshelves/Microbiology/Microbiology_(Bruslind)/15%3A_Phototrophy |
| Ureolysis | Urease (urea amidohydrolase, EC 3.5.1.5) is a nickel-containing enzyme catalyzes the hydrolysis of urea into ammonia and carbamate produced by plants, fungi, and bacteria that [11] |  |
| ⎯⎯⎯⎯⎯⎯⎯⎯⎯⎯⎯⎯⎯⎯⎯⎯⎯⎯⎯⎯⎯⎯⎯⎯⎯⎯⎯⎯⎯⎯⎯⎯⎯⎯⎯⎯⎯⎯⎯⎯⎯⎯⎯⎯⎯⎯⎯⎯⎯⎯⎯⎯⎯⎯⎯⎯⎯⎯⎯⎯⎯⎯⎯⎯⎯⎯⎯⎯⎯ | | |

References

| 1. | Abbasian F, Lockington R, Mallavarapu M, Naidu R (2015) A comprehensive review of aliphatic hydrocarbon biodegradation by Bacteria. Appl Biochem Biotechnol 176(3):670-99. https://doi.org/10.1007/s12010-015-1603-5 |
| --- | --- |
| 2. | Anantharaman K, Breier JA, Sheik CS, Dick GJ (2013) Evidence for hydrogen oxidation and metabolic plasticity in widespread deep-sea sulfur-oxidizing bacteria. Proc Natl Acad Sci USA 110(1):330-5. https://doi.org/10.1073/pnas.1215340110 |
| 3. | Bieberich E (2014) Synthesis, processing, and function of N-glycans in N-glycoproteins. Adv Neurobiol 9:47-70. <https://doi.org/10.1007/978-1-4939-1154-7_3> |
| 4. | Capson-Tojo G, Lin S, Batstone DJ, Hülsen T (2021) Purple phototrophic bacteria are outcompeted by aerobic heterotrophs in the presence of oxygen. Water Res 194:116941. https://doi.org/10.1016/j.watres.2021.116941 |
| 5. | Caspi R, Billington R, Keseler IM, Kothari A, Krummenacker M, Midford PE, Ong WK, Paley S, Subhraveti P, Karp PD (2020) The MetaCyc database of metabolic pathways and enzymes - a 2019 update. Nucleic Acids Res 48(D1):D445-D453. https://doi.org/10.1093/nar/gkz862 |
| 6. | Cheng Y, Elrys AS, Merwad AM, Zhang H, Chen Z, Zhang J, Cai Z, Müller C (2022) Global patterns and drivers of soil dissimilatory nitrate reduction to ammonium. Environ Sci Technol 56(6):3791-3800. https://doi.org/10.1021/acs.est.1c07997 |
| 7. | Chistoserdova L, Kalyuzhnaya MG, Lidstrom ME (2009) The expanding world of methylotrophic metabolism. Annu Rev Microbiol 63:477-99. https://doi.org/10.1146/annurev.micro.091208.073600 |
| 8. | Douglas GM, Maffei VJ, Zaneveld JR, Yurgel SN, Brown JR, Taylor CM, Huttenhower C, Langille MGI (2020) PICRUSt2 for prediction of metagenome functions. Nat Biotechnol 38:685-688. <https://doi.org/10.1038/s41587-020-0548-6> |
| 9. | George DM, Vincent AS, Mackey HR (2020) An overview of anoxygenic phototrophic bacteria and their applications in environmental biotechnology for sustainable Resource recovery. Biotechnol Rep (Amst) 28:e00563. https://doi.org/10.1016/j.btre.2020.e00563 |
| 10. | Ha PT, Lindemann SR, Shi L, Dohnalkova AC, Fredrickson JK, Madigan MT, Beyenal H (2017) Syntrophic anaerobic photosynthesis via direct interspecies electron transfer. Nat Commun 8:13924. https://doi.org/10.1038/ncomms13924 |
| 11. | Kappaun K, Piovesan AR, Carlini CR, Ligabue-Braun R (2018) Ureases: Historical aspects, catalytic, and non-catalytic properties - A review. J Adv Res. 13:3-17. <https://doi.org/10.1016/j.jare.2018.05.010> |
| 12. | Kolb S (2009) Aerobic methanol-oxidizing bacteria in soil. FEMS Microbiol Lett 300(1):1-10. <https://doi.org/10.1111/j.1574-6968.2009.01681.x> |
| 13. | Kraft B, Strous M, Tegetmeyer HE (2011) Microbial nitrate respiration-genes, enzymes and environmental distribution. J Biotechnol 155(1):104-17. https://doi.org/10.1016/j.jbiotec.2010.12.025 |
| 14. | Louca S, Parfrey LW, Doebeli M (2016) Decoupling function and taxonomy in the global ocean microbiome. Science 353:1272-1277. https://doi.org/10.1126/science.aaf4507 |
| 15. | Martínez-Espinosa C, Sauvage S, Al Bitar A, Green PA, Vörösmarty CJ, Sánchez-Pérez JM (2021) Denitrification in wetlands: A review towards a quantification at global scale. Sci Total Environ 754:142398. https://doi.org/10.1016/j.scitotenv.2020.142398 |
| 16. | Nikel PI, Pérez-Pantoja D, de Lorenzo V (2013) Why are chlorinated pollutants so difficult to degrade aerobically? Redox stress limits 1,3-dichloroprop-1-ene metabolism by *Pseudomonas pavonaceae*. Philos Trans R Soc Lond B Biol Sci 368(1616):20120377. <https://doi.org/10.1098/rstb.2012.0377> |
| 17. | Pittard J, Yang J (2008) Biosynthesis of the aromatic aminio acids. EcoSal Plus 3(1). <https://doi.org/10.1128/ecosalplus.3.6.1.8> |
| 18. | Ramond JB, Jordaan K, Díez B, Heinzelmann SM, Cowan DA (2022) Microbial Biogeochemical Cycling of Nitrogen in Arid Ecosystems. Microbiol Mol Biol Rev 86(2):e0010921. https://doi.org/10.1128/mmbr.00109-21 |
| 19. | Roy S, Liu W, Nandety RS, Crook A, Mysore KS, Pislariu CI, Frugoli J, Dickstein R, Udvardi MK (2020) Celebrating 20 years of genetic discoveries in legume nodulation and symbiotic nitrogen fixation. Plant Cell 32(1):15-41. https://doi.org/10.1105/tpc.19.00279 |
| 20. | Sepp SK, Vasar M, Davison J, Oja J, Anslan S, Al-Quraishy S, Bahram M, Bueno CG, Cantero JJ, Fabiano EC, Decocq G, Drenkhan R, Fraser L, Garibay Oriel R, Hiiesalu I, Koorem K, Kõljalg U, Moora M, Mucina L, Öpik M, Põlme S, Pärtel M, Phosri C, Semchenko M, Vahter T, Vasco Palacios AM, Tedersoo L, Zobel M (2023) Global diversity and distribution of nitrogen-fixing bacteria in the soil. Front Plant Sci 14:1100235. <https://doi.org/10.3389/fpls.2023.1100235> |
| 21. | [Yu S, Fiskesund R (2006) **The anhydrofructose pathway and its possible role in stress response and signaling.** Biochim Biophys Acta 1760(9):1314-22.](http://www.ncbi.nlm.nih.gov/pubmed/16822618)  <https://doi.org/10.1016/j.bbagen.2006.05.007> |
