# Supplementary material for: Spatial and Temporal Shifts of Endophytic Bacteria in Conifer Seedlings of Abies religiosa (Kunth) Schltdl. & Cham
Source: Microb Ecol. 2024 Jul 3;87:90. doi: 10.1007/s00248-024-02398-9 (PMC11222277; doi:10.1007/s00248-024-02398-9)
Supplement: Supplementary file 6 [file 248_2024_2398_MOESM5_ESM.docx]

**Table S1.** Metadata of metabarcoding libraries and DNA quantification with Quant-iT PicoGreen dsDNA Assay Kit (Invitrogen, Waltham, MA) on a Nanodrop™ 3300 Fluorometer (Thermo Fisher Scientific, Waltham, MA), previous normalization. Samples with different barcode sequences were pooled together.

| **SampleID** | **BarcodeSequence** | **LinkerPrimerSequence** | **Part** | **Plot** | **Time** | **DNA concentration ng/ml** |
| --- | --- | --- | --- | --- | --- | --- |
| A1.1.6.A1 | TAAGGCGATAGATCGC | GACTACHVGGGTATCTAATCC | Aereo | A1 | 1month | 2423.0 |
| A1.4.3.A | TAAGGCGATATCCTCT | GACTACHVGGGTATCTAATCC | Aereo | A1 | 1month | 3600.0 |
| A1.5.6.A1 | TAAGGCGAGTAAGGAG | GACTACHVGGGTATCTAATCC | Aereo | A1 | 1month | 2963.0 |
| A1.8.7.A1 | TAAGGCGAAAGGAGTA | GACTACHVGGGTATCTAATCC | Aereo | A1 | 1month | 1473.0 |
| A2.1.2.A1 | CGTACTAGTAGATCGC | GACTACHVGGGTATCTAATCC | Aereo | A2 | 1month | 2490.0 |
| A2.2.5.A1 | CGTACTAGTATCCTCT | GACTACHVGGGTATCTAATCC | Aereo | A2 | 1month | 1396.0 |
| A2.3.3.A1 | CGTACTAGGTAAGGAG | GACTACHVGGGTATCTAATCC | Aereo | A2 | 1month | 866.0 |
| A2.9.3.A | CGTACTAGAAGGAGTA | GACTACHVGGGTATCTAATCC | Aereo | A2 | 1month | 5400.0 |
| A3.2.9.A1 | AGGCAGAATAGATCGC | GACTACHVGGGTATCTAATCC | Aereo | A3 | 1month | 2693.0 |
| A3.4.5.A | AGGCAGAATATCCTCT | GACTACHVGGGTATCTAATCC | Aereo | A3 | 1month | 2116.0 |
| A3.6.7.A | AGGCAGAAGTAAGGAG | GACTACHVGGGTATCTAATCC | Aereo | A3 | 1month | 2933.0 |
| A3.8.1.A | AGGCAGAAAAGGAGTA | GACTACHVGGGTATCTAATCC | Aereo | A3 | 1month | 2553.0 |
| B1.2.7.As | TCCTGAGCTAGATCGC | GACTACHVGGGTATCTAATCC | Aereo | B1 | 1month | 2120.0 |
| B1.4.6.As | TCCTGAGCTATCCTCT | GACTACHVGGGTATCTAATCC | Aereo | B1 | 1month | 2856.0 |
| B2.1.7.A1 | TCCTGAGCGTAAGGAG | GACTACHVGGGTATCTAATCC | Aereo | B2 | 1month | 1966.0 |
| B2.10.10.A | GGACTCCTAAGGAGTA | GACTACHVGGGTATCTAATCC | Aereo | B2 | 1month | 9480.0 |
| B2.4.9.A | TCCTGAGCAAGGAGTA | GACTACHVGGGTATCTAATCC | Aereo | B2 | 1month | 1253.0 |
| B2.4.9.Ag | GGACTCCTTAGATCGC | GACTACHVGGGTATCTAATCC | Aereo | B2 | 1month | 1203.0 |
| B2.5.2.A | GGACTCCTTATCCTCT | GACTACHVGGGTATCTAATCC | Aereo | B2 | 1month | 1866.0 |
| B2.8.3.A | GGACTCCTGTAAGGAG | GACTACHVGGGTATCTAATCC | Aereo | B2 | 1month | 1790.0 |
| B3.2.7.A | TAGGCATGTAGATCGC | GACTACHVGGGTATCTAATCC | Aereo | B3 | 1month | 2066.0 |
| B3.4.1.A | TAGGCATGTATCCTCT | GACTACHVGGGTATCTAATCC | Aereo | B3 | 1month | 4183.0 |
| B3.5.2.A | TAGGCATGGTAAGGAG | GACTACHVGGGTATCTAATCC | Aereo | B3 | 1month | 1640.0 |
| B3.8.3.A | TAGGCATGAAGGAGTA | GACTACHVGGGTATCTAATCC | Aereo | B3 | 1month | 1626.0 |
| B3.9.9.A | CTCTCTACTAGATCGC | GACTACHVGGGTATCTAATCC | Aereo | B3 | 1month | 2130.0 |
| A1.1.6.R1 | TAAGGCGACTCTCTAT | GACTACHVGGGTATCTAATCC | Roots | A1 | 1month | 4560.0 |
| A1.4.3.R | TAAGGCGAAGAGTAGA | GACTACHVGGGTATCTAATCC | Roots | A1 | 1month | 3910.0 |
| A1.5.6.R1 | TAAGGCGAACTGCATA | GACTACHVGGGTATCTAATCC | Roots | A1 | 1month | 2183.0 |
| A1.8.7.R1 | TAAGGCGACTAAGCCT | GACTACHVGGGTATCTAATCC | Roots | A1 | 1month | 1973.0 |
| A2.1.2.R1 | CGTACTAGCTCTCTAT | GACTACHVGGGTATCTAATCC | Roots | A2 | 1month | 2146.0 |
| A2.2.5.R1 | CGTACTAGAGAGTAGA | GACTACHVGGGTATCTAATCC | Roots | A2 | 1month | 4820.0 |
| A2.3.3.R1 | CGTACTAGACTGCATA | GACTACHVGGGTATCTAATCC | Roots | A2 | 1month | 3366.0 |
| A2.9.3.R | CGTACTAGCTAAGCCT | GACTACHVGGGTATCTAATCC | Roots | A2 | 1month | 1025.0 |
| A3.2.9.R1 | AGGCAGAACTCTCTAT | GACTACHVGGGTATCTAATCC | Roots | A3 | 1month | 3420.0 |
| A3.4.5.R | AGGCAGAAAGAGTAGA | GACTACHVGGGTATCTAATCC | Roots | A3 | 1month | 1220.0 |
| A3.6.7.R | AGGCAGAAACTGCATA | GACTACHVGGGTATCTAATCC | Roots | A3 | 1month | 3633.0 |
| A3.8.1.R | AGGCAGAACTAAGCCT | GACTACHVGGGTATCTAATCC | Roots | A3 | 1month | 1996.0 |
| B1.2.7.Rs | TCCTGAGCCTCTCTAT | GACTACHVGGGTATCTAATCC | Roots | B1 | 1month | 1533.0 |
| B1.4.6.Rs | TCCTGAGCAGAGTAGA | GACTACHVGGGTATCTAATCC | Roots | B1 | 1month | 3060.0 |
| B2.1.7.R1 | TCCTGAGCACTGCATA | GACTACHVGGGTATCTAATCC | Roots | B2 | 1month | 953.3 |
| B2.10.10.R | GGACTCCTCTAAGCCT | GACTACHVGGGTATCTAATCC | Roots | B2 | 1month | 1493.0 |
| B2.4.9.R | TCCTGAGCCTAAGCCT | GACTACHVGGGTATCTAATCC | Roots | B2 | 1month | 1943.0 |
| B2.4.9.Rg | GGACTCCTCTCTCTAT | GACTACHVGGGTATCTAATCC | Roots | B2 | 1month | 1370.0 |
| B2.5.2.R | GGACTCCTAGAGTAGA | GACTACHVGGGTATCTAATCC | Roots | B2 | 1month | 3336.0 |
| B2.8.3.R | GGACTCCTACTGCATA | GACTACHVGGGTATCTAATCC | Roots | B2 | 1month | 1880.0 |
| B3.2.7.R | TAGGCATGCTCTCTAT | GACTACHVGGGTATCTAATCC | Roots | B3 | 1month | 2753.0 |
| B3.4.1.R | TAGGCATGAGAGTAGA | GACTACHVGGGTATCTAATCC | Roots | B3 | 1month | 2043.0 |
| B3.5.2.R | TAGGCATGACTGCATA | GACTACHVGGGTATCTAATCC | Roots | B3 | 1month | 1520.0 |
| B3.8.3.R | TAGGCATGCTAAGCCT | GACTACHVGGGTATCTAATCC | Roots | B3 | 1month | 1123.0 |
| B3.9.9.R | CTCTCTACCTCTCTAT | GACTACHVGGGTATCTAATCC | Roots | B3 | 1month | 1470.0 |
| A1.8.7.35aA | TAGGCATGCTCTCTAT | GACTACHVGGGTATCTAATCC | Aereo | A1 | 5months | 918.0 |
| A1.8.7.35bA | TAGGCATGGTAAGGAG | GACTACHVGGGTATCTAATCC | Aereo | A1 | 5months | 1350.0 |
| A2.1.2.35A | TAGGCATGTATCCTCT | GACTACHVGGGTATCTAATCC | Aereo | A2 | 5months | 1473.3 |
| A2.2.5.35aA | TAGGCATGTAGATCGC | GACTACHVGGGTATCTAATCC | Aereo | A2 | 5months | 1120.0 |
| A2.2.5.35bA | GGACTCCTCTAAGCCT | GACTACHVGGGTATCTAATCC | Aereo | A2 | 5months | 842.0 |
| A2.2.5.35cA | TAGGCATGACTGCATA | GACTACHVGGGTATCTAATCC | Aereo | A2 | 5months | 1093.3 |
| A2.5.6.36A | TCCTGAGCAGAGTAGA | GACTACHVGGGTATCTAATCC | Aereo | A2 | 5months | 1473.3 |
| A2.9.3.35A | TCCTGAGCACTGCATA | GACTACHVGGGTATCTAATCC | Aereo | A2 | 5months | 712.7 |
| A3.2.9.35aA | TCCTGAGCGTAAGGAG | GACTACHVGGGTATCTAATCC | Aereo | A3 | 5months | 714.7 |
| A3.2.9.35bA | TCCTGAGCCTAAGCCT | GACTACHVGGGTATCTAATCC | Aereo | A3 | 5months | 1946.7 |
| A3.2.9.35cA | TCCTGAGCAAGGAGTA | GACTACHVGGGTATCTAATCC | Aereo | A3 | 5months | 733.3 |
| A3.2.9.35dA | TAGGCATGAAGGAGTA | GACTACHVGGGTATCTAATCC | Aereo | A3 | 5months | 720.7 |
| A3.2.9.36A | TAGGCATGCTAAGCCT | GACTACHVGGGTATCTAATCC | Aereo | A3 | 5months | 824.7 |
| A3.4.6.36A | CTCTCTACGTAAGGAG | GACTACHVGGGTATCTAATCC | Aereo | A3 | 5months | 890.7 |
| B1.2.4.35aA | CTCTCTACAGAGTAGA | GACTACHVGGGTATCTAATCC | Aereo | B1 | 5months | 1400.0 |
| B1.2.4.35bA | GGACTCCTACTGCATA | GACTACHVGGGTATCTAATCC | Aereo | B1 | 5months | 792.7 |
| B2.1.7.aA | GGACTCCTGTAAGGAG | GACTACHVGGGTATCTAATCC | Aereo | B2 | 5months | 960.0 |
| B2.1.7.bA | GGACTCCTTATCCTCT | GACTACHVGGGTATCTAATCC | Aereo | B2 | 5months | 1056.7 |
| B2.10.10.aA | GGACTCCTCTCTCTAT | GACTACHVGGGTATCTAATCC | Aereo | B2 | 5months | 699.3 |
| B2.10.10.bA | TAGGCATGAGAGTAGA | GACTACHVGGGTATCTAATCC | Aereo | B2 | 5months | 943.3 |
| B2.4.9.A | GGACTCCTAAGGAGTA | GACTACHVGGGTATCTAATCC | Aereo | B2 | 5months | 417.3 |
| B3.2.3.32A | CTCTCTACCTCTCTAT | GACTACHVGGGTATCTAATCC | Aereo | B3 | 5months | 822.7 |
| B3.2.7.aA | CTCTCTACTATCCTCT | GACTACHVGGGTATCTAATCC | Aereo | B3 | 5months | 1350.0 |
| B3.2.7.bA | CTCTCTACACTGCATA | GACTACHVGGGTATCTAATCC | Aereo | B3 | 5months | 806.0 |
| B3.9.9.35A | GGACTCCTAGAGTAGA | GACTACHVGGGTATCTAATCC | Aereo | B3 | 5months | 707.3 |
| B3.9.9.35aA | CTCTCTACTAGATCGC | GACTACHVGGGTATCTAATCC | Aereo | B3 | 5months | 743.3 |
| B3.9.9.35cA | GGACTCCTTAGATCGC | GACTACHVGGGTATCTAATCC | Aereo | B3 | 5months | 976.7 |
| A1.8.7.35a | CTCTCTACTATCCTCT | GACTACHVGGGTATCTAATCC | Rhizoplane | A1 | 5months | 3306.0 |
| A1.8.7.b | CTCTCTACAGAGTAGA | GACTACHVGGGTATCTAATCC | Rhizoplane | A1 | 5months | 3070.0 |
| A2.1.2. | CTCTCTACGTAAGGAG | GACTACHVGGGTATCTAATCC | Rhizoplane | A2 | 5months | 12000.0 |
| A2.2.5.a | CTCTCTACACTGCATA | GACTACHVGGGTATCTAATCC | Rhizoplane | A2 | 5months | 2480.0 |
| A2.2.5.b | CTCTCTACAAGGAGTA | GACTACHVGGGTATCTAATCC | Rhizoplane | A2 | 5months | 1550.0 |
| A2.2.5.c | CTCTCTACCTAAGCCT | GACTACHVGGGTATCTAATCC | Rhizoplane | A2 | 5months | 1282.0 |
| A2.6.5. | CAGAGAGGCTCTCTAT | GACTACHVGGGTATCTAATCC | Rhizoplane | A2 | 5months | 4116.0 |
| A2.9.3. | CAGAGAGGTAGATCGC | GACTACHVGGGTATCTAATCC | Rhizoplane | A2 | 5months | 2416.0 |
| A3.2.9.a | CAGAGAGGTATCCTCT | GACTACHVGGGTATCTAATCC | Rhizoplane | A3 | 5months | 2610.0 |
| A3.2.9.b | CAGAGAGGAGAGTAGA | GACTACHVGGGTATCTAATCC | Rhizoplane | A3 | 5months | 5160.0 |
| A3.2.9.c | CAGAGAGGGTAAGGAG | GACTACHVGGGTATCTAATCC | Rhizoplane | A3 | 5months | 680.0 |
| A3.2.9.d | CAGAGAGGACTGCATA | GACTACHVGGGTATCTAATCC | Rhizoplane | A3 | 5months | 6526.7 |
| A3.2.9.e | CAGAGAGGAAGGAGTA | GACTACHVGGGTATCTAATCC | Rhizoplane | A3 | 5months | 1770.0 |
| A3.4.6. | CAGAGAGGCTAAGCCT | GACTACHVGGGTATCTAATCC | Rhizoplane | A3 | 5months | 2236.0 |
| B1.1.7.a | GCTACGCTTATCCTCT | GACTACHVGGGTATCTAATCC | Rhizoplane | B1 | 5months | 168.0 |
| B1.1.7.b | GCTACGCTAGAGTAGA | GACTACHVGGGTATCTAATCC | Rhizoplane | B1 | 5months | 1840.0 |
| B1.2.4.a | GCTACGCTTAGATCGC | GACTACHVGGGTATCTAATCC | Rhizoplane | B1 | 5months | 1993.0 |
| B1.2.4.b | GCTACGCTCTCTCTAT | GACTACHVGGGTATCTAATCC | Rhizoplane | B1 | 5months | 8346.7 |
| B2.10.10.a | GCTACGCTACTGCATA | GACTACHVGGGTATCTAATCC | Rhizoplane | B2 | 5months | 1193.0 |
| B2.10.10.b | GCTACGCTAAGGAGTA | GACTACHVGGGTATCTAATCC | Rhizoplane | B2 | 5months | 1246.0 |
| B2.4.9. | GCTACGCTGTAAGGAG | GACTACHVGGGTATCTAATCC | Rhizoplane | B2 | 5months | 6786.7 |
| B3.2.3.32 | CGAGGCTGGTAAGGAG | GACTACHVGGGTATCTAATCC | Rhizoplane | B3 | 5months | 1123.0 |
| B3.2.7.a | GCTACGCTCTAAGCCT | GACTACHVGGGTATCTAATCC | Rhizoplane | B3 | 5months | 1343.0 |
| B3.2.7.b | CGAGGCTGTAGATCGC | GACTACHVGGGTATCTAATCC | Rhizoplane | B3 | 5months | 1393.0 |
| B3.9.9.a | CGAGGCTGCTCTCTAT | GACTACHVGGGTATCTAATCC | Rhizoplane | B3 | 5months | 8526.7 |
| B3.9.9.b | CGAGGCTGTATCCTCT | GACTACHVGGGTATCTAATCC | Rhizoplane | B3 | 5months | 7593.3 |
| B3.9.9.c | CGAGGCTGAGAGTAGA | GACTACHVGGGTATCTAATCC | Rhizoplane | B3 | 5months | 8240.0 |
| A1.8.7.aR | CGTACTAGAAGGAGTA | GACTACHVGGGTATCTAATCC | Roots | A1 | 5months | 2270.0 |
| A1.8.7.bR | AGGCAGAACTCTCTAT | GACTACHVGGGTATCTAATCC | Roots | A1 | 5months | 1166.7 |
| A2.1.2.R | CGTACTAGCTAAGCCT | GACTACHVGGGTATCTAATCC | Roots | A2 | 5months | 751.3 |
| A2.2.5.aR | CGTACTAGACTGCATA | GACTACHVGGGTATCTAATCC | Roots | A2 | 5months | 1196.7 |
| A2.2.5.bR | CGTACTAGGTAAGGAG | GACTACHVGGGTATCTAATCC | Roots | A2 | 5months | 841.3 |
| A2.2.5.cR | AGGCAGAATATCCTCT | GACTACHVGGGTATCTAATCC | Roots | A2 | 5months | 706.0 |
| A2.6.5.36R | TAAGGCGATAGATCGC | GACTACHVGGGTATCTAATCC | Roots | A2 | 5months | 636.0 |
| A2.9.3.35R | TAAGGCGATATCCTCT | GACTACHVGGGTATCTAATCC | Roots | A2 | 5months | 1493.3 |
| A3.2.9.35aR | TAAGGCGACTCTCTAT | GACTACHVGGGTATCTAATCC | Roots | A3 | 5months | 820.0 |
| A3.2.9.35cR | TAAGGCGAAGAGTAGA | GACTACHVGGGTATCTAATCC | Roots | A3 | 5months | 756.7 |
| A3.2.9.b35R | TAAGGCGAGTAAGGAG | GACTACHVGGGTATCTAATCC | Roots | A3 | 5months | 2090.0 |
| A3.2.9.dR | AGGCAGAAAGAGTAGA | GACTACHVGGGTATCTAATCC | Roots | A3 | 5months | 2540.0 |
| A3.2.9.R | AGGCAGAAGTAAGGAG | GACTACHVGGGTATCTAATCC | Roots | A3 | 5months | 790.7 |
| A3.4.6.R | TCCTGAGCCTCTCTAT | GACTACHVGGGTATCTAATCC | Roots | A3 | 5months | 1086.7 |
| B1.2.4.aR | TCCTGAGCTAGATCGC | GACTACHVGGGTATCTAATCC | Roots | B1 | 5months | 632.7 |
| B1.2.4.bR | CGTACTAGTATCCTCT | GACTACHVGGGTATCTAATCC | Roots | B1 | 5months | 1160.0 |
| B2.1.7.aR | CGTACTAGCTCTCTAT | GACTACHVGGGTATCTAATCC | Roots | B2 | 5months | 2093.3 |
| B2.1.7.bR | TAAGGCGACTAAGCCT | GACTACHVGGGTATCTAATCC | Roots | B2 | 5months | 1500.0 |
| B2.10.10.bR | AGGCAGAATAGATCGC | GACTACHVGGGTATCTAATCC | Roots | B2 | 5months | 1266.7 |
| B2.4.9.R | CGTACTAGAGAGTAGA | GACTACHVGGGTATCTAATCC | Roots | B2 | 5months | 1086.7 |
| B23.2.7.aR | AGGCAGAACTAAGCCT | GACTACHVGGGTATCTAATCC | Roots | B2 | 5months | 1360.0 |
| B3.2.3.R | AGGCAGAAAAGGAGTA | GACTACHVGGGTATCTAATCC | Roots | B3 | 5months | 1193.3 |
| B3.2.7.bR | TCCTGAGCTATCCTCT | GACTACHVGGGTATCTAATCC | Roots | B3 | 5months | 1306.7 |
| B3.9.9.35cR | TAAGGCGAACTGCATA | GACTACHVGGGTATCTAATCC | Roots | B3 | 5months | 963.3 |
| B3.9.9.bR | CGTACTAGTAGATCGC | GACTACHVGGGTATCTAATCC | Roots | B3 | 5months | 1123.3 |
| B3.9.9.dR | AGGCAGAAACTGCATA | GACTACHVGGGTATCTAATCC | Roots | B3 | 5months | 1193.3 |
