# Supplementary material for: Spatial and Temporal Shifts of Endophytic Bacteria in Conifer Seedlings of Abies religiosa (Kunth) Schltdl. & Cham
Source: Microb Ecol. 2024 Jul 3;87:90. doi: 10.1007/s00248-024-02398-9 (PMC11222277; doi:10.1007/s00248-024-02398-9)
Supplement: Supplementary file 8 [file 248_2024_2398_MOESM7_ESM.docx]

**Table S3** Permutational multivariate analysis of variance (perMANOVA) to determine the effect of plant part (rhizoplane, roots and aerial parts) and time (one and five months) on the bacterial phyla, genera and amplicon sequence variants (ASVs), putative metabolic pathways at level 3 of MetaCyc as determined with PICRUSt2, and putative functional groups as determined with FAPROTAX. Permutation multivariate analysis of dispersion (PERMDISP) was used to determine if the dispersion within the compared treatments was significant

| ⎯⎯⎯⎯⎯⎯⎯⎯⎯⎯⎯⎯⎯⎯⎯⎯⎯⎯⎯⎯⎯⎯⎯⎯⎯⎯⎯⎯⎯⎯⎯⎯⎯⎯⎯⎯⎯⎯⎯⎯ | | | | | | | | | |  |
| --- | --- | --- | --- | --- | --- | --- | --- | --- | --- | --- |
|  | | perMANOVA | | | PERMDISP | | | | |  |
|  | | ⎯⎯⎯⎯⎯⎯⎯⎯⎯⎯ | | | ⎯⎯⎯⎯⎯⎯⎯ | | | | |  |
|  |  | F value | R^2^ | *p* value | F value | | | *p* value | |  |
| ⎯⎯⎯⎯⎯⎯⎯⎯⎯⎯⎯⎯⎯⎯⎯⎯⎯⎯⎯⎯⎯⎯⎯⎯⎯⎯⎯⎯⎯⎯⎯⎯⎯⎯⎯⎯⎯⎯⎯⎯ | | | | | | | | | |  |
| Bacterial phyla | | | | | | | | | |  |
| ⎯⎯⎯⎯⎯⎯⎯⎯⎯⎯⎯⎯⎯⎯⎯⎯⎯⎯⎯⎯⎯⎯⎯⎯⎯⎯⎯⎯⎯⎯⎯⎯⎯⎯⎯⎯⎯⎯⎯⎯ | | | | | | | | | |  |
| After 1 month | Aerial parts vs roots | 10.1 | 17 | **< 0.001 ^a^** | | 1.9 | 0.206 | | |  |
| After 5 months | Plant compartments | 24.6 | 39 | **< 0.001** |  | | |  | |  |
|  | Aerial parts vs rhizoplane | 25.8 | 33 | **< 0.001** | 0.3 | | | 0.564 | |  |
|  | Aerial parts vs roots | 10.8 | 18 | **< 0.001** | 0.6 | | | 0.450 | |  |
|  | Rhizoplane vs roots | 6.5 | 11 | **< 0.001** | 1.1 | | | 0.318 | |  |
| Aerial parts | One month vs five months | 1.6 | 3 | **0.027** | 0.1 | | | 0.983 | |  |
| Roots | One month vs five months | 3.2 | 6 | **< 0.001** | 0.7 | | | 0.399 | |  |
| ⎯⎯⎯⎯⎯⎯⎯⎯⎯⎯⎯⎯⎯⎯⎯⎯⎯⎯⎯⎯⎯⎯⎯⎯⎯⎯⎯⎯⎯⎯⎯⎯⎯⎯⎯⎯⎯⎯⎯⎯ | | | | | | | | | |  |
| Bacterial genera | | | | | | | | | |  |
| ⎯⎯⎯⎯⎯⎯⎯⎯⎯⎯⎯⎯⎯⎯⎯⎯⎯⎯⎯⎯⎯⎯⎯⎯⎯⎯⎯⎯⎯⎯⎯⎯⎯⎯⎯⎯⎯⎯⎯⎯ | | | | | | | | | |  |
| After 1 month | Aerial parts vs roots | 4.0 | 8 | **< 0.001** | 0.5 | | | 0.501 | |  |
| After 5 months | Plant compartments | 9.8 | 20 | **< 0.001** |  | | |  | |  |
|  | Aerial parts vs rhizoplane | 11.3 | 18 | **< 0.001** | 2.2 | | | 0.172 | |  |
|  | Aerial parts vs roots | 4.5 | 8 | **< 0.001** | 10.1 | | | **0.019** | |  |
|  | Rhizoplane vs roots | 4.5 | 8 | **< 0.001** | 0.5 | | | 0.488 | |  |
| Aerial parts | One month vs five months | 1.6 | 3 | **< 0.001** | 0.6 | | | 0.463 | |  |
| Roots | One month vs five months | 3.0 | 6 | **< 0.001** | 8.5 | | | **0.008** | |  |
| ⎯⎯⎯⎯⎯⎯⎯⎯⎯⎯⎯⎯⎯⎯⎯⎯⎯⎯⎯⎯⎯⎯⎯⎯⎯⎯⎯⎯⎯⎯⎯⎯⎯⎯⎯⎯⎯⎯⎯⎯ | | | | | | | | | |  |
| Bacterial amplicon sequence variants (ASVs) | | | | | | | | | |  |
| ⎯⎯⎯⎯⎯⎯⎯⎯⎯⎯⎯⎯⎯⎯⎯⎯⎯⎯⎯⎯⎯⎯⎯⎯⎯⎯⎯⎯⎯⎯⎯⎯⎯⎯⎯⎯⎯⎯⎯⎯ | | | | | | | | | |  |
| After 1 month | Aerial parts vs roots | 1.5 | 3 | **< 0.001** | 1.2 | | | 0.328 | |  |
| After 5 months | Plant compartments | 1.9 | 5 | **< 0.001** |  | | |  | |  |
|  | Aerial parts vs rhizoplane | 2.6 | 5 | **< 0.001** | 77.4 | | | **<0.001** | |  |
|  | Aerial parts vs roots | 1.5 | 3 | **< 0.001** | 24.3 | | | **0.003** | |  |
|  | Rhizoplane vs roots | 1.7 | 3 | **< 0.001** | 19.0 | | | **<0.001** | |  |
| Aerial parts | One month vs five months | 1.1 | 2 | **< 0.001** | 1.3 | | | 0.277 | |  |
| Roots | One month vs five months | 1.6 | 2 | **< 0.001** | 8.1 | | | **0.021** | |  |
| ⎯⎯⎯⎯⎯⎯⎯⎯⎯⎯⎯⎯⎯⎯⎯⎯⎯⎯⎯⎯⎯⎯⎯⎯⎯⎯⎯⎯⎯⎯⎯⎯⎯⎯⎯⎯⎯⎯⎯ | | | | | | | | | |  |
| Putative metabolic pathways at level 3 of MetaCyc | | | | | | | | | | |
| ⎯⎯⎯⎯⎯⎯⎯⎯⎯⎯⎯⎯⎯⎯⎯⎯⎯⎯⎯⎯⎯⎯⎯⎯⎯⎯⎯⎯⎯⎯⎯⎯⎯⎯⎯⎯⎯⎯⎯⎯ | | | | | | | | | | |
| After 1 month | Aerial parts vs roots | 30.4 | 37 | **< 0.001** | 6.7 | | | **0.017** | |  |
| After 5 months | Plant compartments | 25.5 | 40 | **< 0.001** |  | | |  | |  |
|  | Aerial parts vs rhizoplane | 47.0 | 48 | **< 0.001** | 2.6 | | | 0.117 | |  |
|  | Aerial parts vs roots | 21.9 | 30 | **< 0.001** | 0.5 | | | 0.622 | |  |
|  | Rhizoplane vs roots | 6.7 | 11 | **< 0.001** | 3.0 | | | 0.128 | |  |
| Aerial parts | One month vs five months | 4.5 | 8 | **0.003** | 1.1 | | | 0.239 | |  |
| Roots | One month vs five months | 11.0 | 18 | **< 0.001** | 0.6 | | | 0.474 | |  |
| ⎯⎯⎯⎯⎯⎯⎯⎯⎯⎯⎯⎯⎯⎯⎯⎯⎯⎯⎯⎯⎯⎯⎯⎯⎯⎯⎯⎯⎯⎯⎯⎯⎯⎯⎯⎯⎯⎯⎯ | | | | | | | | |  |  |

**Table S3** Continued

| ⎯⎯⎯⎯⎯⎯⎯⎯⎯⎯⎯⎯⎯⎯⎯⎯⎯⎯⎯⎯⎯⎯⎯⎯⎯⎯⎯⎯⎯⎯⎯⎯⎯⎯⎯⎯⎯⎯⎯⎯ | | | | | | | | |
| --- | --- | --- | --- | --- | --- | --- | --- | --- |
| Putative functional groups | | | | | | | | |
| ⎯⎯⎯⎯⎯⎯⎯⎯⎯⎯⎯⎯⎯⎯⎯⎯⎯⎯⎯⎯⎯⎯⎯⎯⎯⎯⎯⎯⎯⎯⎯⎯⎯⎯⎯⎯⎯⎯⎯⎯ | | | | | | | | |
| After 1 month | Aerial parts vs roots | 7.8 | 14.0 | **< 0.001** | 0.5 | 0.567 | |  |
| After 5 months | Plant compartments | 10.3 | 22.0 | **< 0.001** |  |  | |  |
|  | Aerial parts vs rhizoplane | 16.7 | 25.4 | **< 0.001** | 11.5 | **0.004** | |  |
|  | Aerial parts vs roots | 10.3 | 21.7 | **< 0.001** | 2.0 | 0.197 | |  |
|  | Rhizoplane vs roots | 8.8 | 15.0 | **< 0.001** | 2.0 | 0.188 | |  |
| Aerial parts | One month vs five months | 3.0 | 6.1 | **0.002** | 0.2 | 0.642 | |  |
| Roots | One month vs five months | 2.6 | 4.9 | **0.006** | 0.5 | 0.541 | |  |
| ⎯⎯⎯⎯⎯⎯⎯⎯⎯⎯⎯⎯⎯⎯⎯⎯⎯⎯⎯⎯⎯⎯⎯⎯⎯⎯⎯⎯⎯⎯⎯⎯⎯⎯⎯⎯⎯⎯⎯ | | | | | | |  |  |

^a^ Values in bold are significant at *p*<0.05
