# Supplementary material for: Spatial and Temporal Shifts of Endophytic Bacteria in Conifer Seedlings of Abies religiosa (Kunth) Schltdl. & Cham
Source: Microb Ecol. 2024 Jul 3;87:90. doi: 10.1007/s00248-024-02398-9 (PMC11222277; doi:10.1007/s00248-024-02398-9)
Supplement: Supplementary file 10 [file 248_2024_2398_MOESM9_ESM.docx]

**Table S5** The ten most abundant bacterial genera found in the aerial parts, roots and rhizoplane after one and five months of the conifer *Abies religiosa* with some of metabolic capacities they possess.

| ⎯⎯⎯⎯⎯⎯⎯⎯⎯⎯⎯⎯⎯⎯⎯⎯⎯⎯⎯⎯⎯⎯⎯⎯⎯⎯⎯⎯⎯⎯⎯⎯⎯⎯⎯⎯⎯⎯⎯⎯⎯⎯⎯⎯⎯⎯⎯⎯⎯⎯⎯⎯⎯⎯⎯⎯ | | | | |
| --- | --- | --- | --- | --- |
| Endophyte | Host plant they were detected in | Location in plant | Metabolic function that might contribute to plant host development | Author(s) |
| ⎯⎯⎯⎯⎯⎯⎯⎯⎯⎯⎯⎯⎯⎯⎯⎯⎯⎯⎯⎯⎯⎯⎯⎯⎯⎯⎯⎯⎯⎯⎯⎯⎯⎯⎯⎯⎯⎯⎯⎯⎯⎯⎯⎯⎯⎯⎯⎯⎯⎯⎯⎯⎯⎯⎯⎯ | | | | |
| *Pseudomonas*  (T1 A:70.1, R:35.8) ^a^  (T5 A:17.3, R:0.5, Rh:2.8) | Sugarcane (*Saccharum* spp.)  Willow (*Salix sitchensis*)  Pine (Pinus spp.) | Root  Stem  Needle, stem, root | Antifungal activity against sugarcane pathogens  Nitrogen fixation  Biocontrol agents against pine wood nematodes | [19]  [7]  [15] |
|  | Banana (Musa spp.) | Endophyte | Indole acetic acid and siderophores production, P solubilization P, antagonistic against *Fusarium oxysporum* f. sp. *Cubense.* | [16] |
|  |  |  |  |  |
| *Allorhizobium-Neorhizobium-Pararhizobium-Rhizobium*  (T1 A:0.9, R:9.4)  (T5 A:7.3, R:9.0, Rh:6.7) | Duckweeds (Pirodela, Landoltia, Lemna, Wolffia)  Pearlwort (*Colobanthus quitensis*)  *Alkanna tinctoria* L. | Entire plant  Leaves  Root | Plant growth promoting (PGP)  PGP at low temperature  Possible production of alkannins | [3]  [17]  [6] |
| *Rhizobium* | Banana (Musa spp.) | Endophyte | Indole acetic acid and siderophores production, P solubilization P, antagonistic against *Fusarium oxysporum* f. sp. *Cubense.* | [16] |
| *Rhizobium tropici* | Populus | Stem endophyte | Nitrogen fixing | [7] |
|  |  |  |  |  |
| *Streptomyces*  (T1 A:0.04, R:4.5)  (T5 A:0.7, R:12.3, Rh:2.5) | *Arabidopsis thaliana*  Wheat (*Triticum aestivum*)  *Maesa japonica* | Root  Root  Leaves | PGP  PGP and biocontrol against *Fusarium graminearum*  Antimicrobial activity | [25]  [5]  [21] |
|  | Banana (Musa spp.) | Endophyte | Indole acetic acid and siderophores production, P solubilization, antagonistic against *Fusarium oxysporum* f. sp. *Cubense.* | [16] |
|  |  |  |  |  |
| *Mucilaginibacter*  (T1 A:0.04, R:4.5)  (T5 A:0.7, R:12.3, Rh:2.5) | Fall dandelion (*Corzoneroides autumnalis*)  Grass “Kleine Fontane” (*Miscanthus sinensis*) Marama bean (Tylosema esculentum) | Root  Stem  Shoot, root | PGP and salinity stress alleviator  Polysaccharide-producing bacteria  PGP (AIA producer) | [8]  [28]  [4] |
| ⎯⎯⎯⎯⎯⎯⎯⎯⎯⎯⎯⎯⎯⎯⎯⎯⎯⎯⎯⎯⎯⎯⎯⎯⎯⎯⎯⎯⎯⎯⎯⎯⎯⎯⎯⎯⎯⎯⎯⎯⎯⎯⎯⎯⎯⎯⎯⎯⎯⎯⎯⎯⎯⎯⎯⎯ | | | | |

**Table S5** Continued

| ⎯⎯⎯⎯⎯⎯⎯⎯⎯⎯⎯⎯⎯⎯⎯⎯⎯⎯⎯⎯⎯⎯⎯⎯⎯⎯⎯⎯⎯⎯⎯⎯⎯⎯⎯⎯⎯⎯⎯⎯⎯⎯⎯⎯⎯⎯⎯⎯⎯⎯⎯⎯⎯⎯⎯⎯ | | | | |
| --- | --- | --- | --- | --- |
| *Sphingomonas*  (T1 A:1.1, R:2.5)  (T5 A:5.3, R:1.9, Rh:2.4) | *Sedum alfredii*  Tephrosia apollinea | Shoots, roots  Leaves | PGP in Cd contaminated soil  PGP in *Solanum pimpinellifolium* in saline condition; PGP *Solanum lycopersicum* in saline condition | [24]  [13]  [12] |
|  |  |  |  |  |
| *Burkholderia-Caballeronia-Paraburkholderia*  (T1 A:1.3, R:4.0)  (T5 A:1.2, R:1.2, Rh:1.9) | Rice (*Oryza sativa*)  *Atractylodes lancea*  Water yam *(Dioscorea alata)* | Root  Root  Leaves, stem, root | PGP in low N amounts  PGP and improved heat stress tolerance  PGP | [27]  [23]  [14] |
|  |  |  |  |  |
| *Luteibacter*  (T1 A:3.2, R:2.2)  (T5 A:0.5, R:0.7, Rh:1.5) | Tea plant (*Camellia sinensis*)  Amazonian palm (*Astrocaryum sciophilum*)  Mediterranean orchids (Neottia ovata, Serapias vomeracea, Spiranthes spiralis) | Leaves  Leaves  Roots, stem, leaves | Secondary metabolite (theanine) production  Antimicrobial activity  PGP activity | [20]  [2]  [34] |
|  | Banana (Musa spp.) | Endophyte | Indole acetic acid and siderophores production, P solubilization P, antagonistic against *Fusarium oxysporum* f. sp. *Cubense.* | [1] |
|  |  |  |  |  |
| *Flavobacterium*  (T1 A:0.6, R:3.4)  (T5 A:1.0, R:0.3, Rh:1.4) | Maize (*Zea mays*)  Panax ginseng  Rice (Oryza sativa) | Root  Root  Seeds | Possible nitrogen fixative (nifh)  β‐glucosidase producer  PGP (phosphate solubilizers and indole-3-acetic acid producer) | [11]  [10]  [22] |
|  |  |  |  |  |
| *Puia*  (T1 A:0.02, R:0.4)  (T5 A:0.3, R:3.5, Rh:2.2) | Populus tremula  Rice (Oryza sativa) | Root  Root | No reported  No reported | [9]  [18] |
|  |  |  |  |  |
| *Collimonas*  (T1 A:0.01, R:0.2)  (T5 A:0.5, R:3.2, Rh:1.8) | Vascular plants (maize), *Vicia faba* and other | Stem, roots | PGP activity | [26] |
| ⎯⎯⎯⎯⎯⎯⎯⎯⎯⎯⎯⎯⎯⎯⎯⎯⎯⎯⎯⎯⎯⎯⎯⎯⎯⎯⎯⎯⎯⎯⎯⎯⎯⎯⎯⎯⎯⎯⎯⎯⎯⎯⎯⎯⎯⎯⎯⎯⎯⎯⎯⎯⎯⎯⎯⎯ | | | | |

^a^ T1: after one month, A: aerial parts, R: Roots, T5 after five months, Rh: Rhizosphere with values given the relative abundance (%).

References

| 1. | Alibrandi P, Schnell S, Perotto S, Cardinale, M (2020) Diversity and structure of the endophytic bacterial communities associated with three terrestrial orchid species as revealed by 16S rRNA gene metabarcoding. Front Microbiol 11:604964. <https://doi.org/10.3389/FMICB.2020.604964/FULL> |
| --- | --- |
| 2. | Barthélemy M, Elie N, Pellissier L, Wolfender JL, Stien D, Touboul D, Eparvier V (2019) Structural identification of antibacterial lipids from Amazonian palm tree endophytes through the molecular network approach. Int J Mol Sci 20(8):2006. <https://doi.org/10.3390/IJMS20082006> |
| 3. | Bunyoo C, Roongsattham P, Khumwan S, Phonmakham J, Wonnapinij P, Thamchaipenet A (2022) Dynamic alteration of microbial communities of duckweeds from nature to nutrient-deficient condition. Plants 11:2915. <https://doi.org/10.3390/plants11212915> |
| 4. | Chimwamurombe PM, Grönemeyer JL, Reinhold-Hurek B (2016) Isolation and characterization of culturable seed-associated bacterial endophytes from gnotobiotically grown Marama bean seedlings. FEMS Microbiol Ecol 92(6):fiw083. <https://doi.org/10.1093/femsec/fiw083> |
| 5. | Colombo EM, Kunova A, Pizzatti C, Saracchi M, Cortesi P, Pasquali M (2019) Selection of an endophytic *Streptomyces* sp. strain DEF09 from wheat roots as a biocontrol agent against *Fusarium graminearum*. Front. Microbiol 10:2356. doi: <https://doi.org/10.3389/fmicb.2019.02356> |
| 6. | Csorba C, Rodić N, Zhao Y, Antonielli L, Brader G, Vlachou A, Tsiokanos E, Lalaymia I, Declerck S, Papageorgiou VP, Assimopoulou AN, Sessitsch A (2022) Metabolite production in *Alkanna tinctoria* links plant development with the recruitment of individual members of microbiome thriving at the root-soil interface. mSystems 7(5):e0045122. https://doi.org/10.1128/msystems.00451-22 |
| 7. | Doty SL, Dosher MR, Singleton GL, [Moore](https://www.researchgate.net/scientific-contributions/AL-Moore-2086219508?_tp=eyJjb250ZXh0Ijp7ImZpcnN0UGFnZSI6InB1YmxpY2F0aW9uIiwicGFnZSI6InB1YmxpY2F0aW9uIn19) AL, [Van Aken](https://www.researchgate.net/profile/Benoit-Van-Aken?_tp=eyJjb250ZXh0Ijp7ImZpcnN0UGFnZSI6InB1YmxpY2F0aW9uIiwicGFnZSI6InB1YmxpY2F0aW9uIn19) B, [Stettler](https://www.researchgate.net/profile/Reinhard-Stettler?_tp=eyJjb250ZXh0Ijp7ImZpcnN0UGFnZSI6InB1YmxpY2F0aW9uIiwicGFnZSI6InB1YmxpY2F0aW9uIn19) R,  [Strand](https://www.researchgate.net/profile/Stuart-Strand?_tp=eyJjb250ZXh0Ijp7ImZpcnN0UGFnZSI6InB1YmxpY2F0aW9uIiwicGFnZSI6InB1YmxpY2F0aW9uIn19) SE, [Gordon](https://www.researchgate.net/scientific-contributions/MP-Gordon-2043755189?_tp=eyJjb250ZXh0Ijp7ImZpcnN0UGFnZSI6InB1YmxpY2F0aW9uIiwicGFnZSI6InB1YmxpY2F0aW9uIn19) MP (2005) Identification of an endophytic *Rhizobium* in stems of *Populus.* [Symbiosis](https://www.researchgate.net/journal/Symbiosis-0334-5114) 39(1):27-35. <https://doi.org/10.1007/BF03179967> |
| 8. | Fan D, Smith DL (2022) *Mucilaginibacter* sp. K improves growth and induces salt tolerance in nonhost plants *via* multilevel mechanisms. Front Plant Sci 13:938697. https://doi.org/10.3389/fpls.2022.938697 |
| 9. | Fracchia F, Mangeot-Peter L, Jacquot L, Martin F, Veneault-Fourrey C, Deveau A (2021) Colonization of naive roots from *Populus tremula × alba* involves successive waves of Fungi and Bacteria with different trophic abilities. Appl Environ Microbiol 87(6):e02541-20. https://doi.org/10.1128/AEM.02541-20. |
| 10. | Fu Y (2019) Biotransformation of ginsenoside Rb1 to Gyp‐XVII and minor ginsenoside Rg3 by endophytic bacterium *Flavobacterium* sp. GE 32 isolated from *Panax ginseng*. Lett Appl Microbiol 68(2):134-141. <https://doi.org/10.1111/LAM.13090> |
| 11. | Gao JL, Lv FY, Wang XM, Yuan M, Li JW, Wu QY, Sun JG (2015) *Flavobacterium endophyticum* sp. nov, a nifH gene-harbouring endophytic bacterium isolated from maize root. Int J Syst Evol Microbiol 65(11):3900-3904. https://doi.org/10.1099/ijsem.0.000513 |
| 12. | Halo BA, Khan AL, Waqas M, Al-Harrasi A, Hussain J, Ali L, Adnan M, Lee IJ (2015) Endophytic bacteria (*Sphingomonas* sp. LK11) and gibberellin can improve *Solanum lycopersicum* growth and oxidative stress under salinity. J Plant Interact 10(1):117-125. <https://doi.org/10.1080/17429145.2015.1033659> |
| 13. | Khan AL, Waqas M, Asaf S, Kamran M, Shahzad R, Bilal S, Khan MA, Kang, SM, Kim YH, Yun BW, Al-Rawahi A, Al-Harrasi A, Lee IJ (2017) Plant growth-promoting endophyte *Sphingomonas* sp. LK11 alleviates salinity stress in *Solanum pimpinellifolium*. Environ Exp Bot 133:58-69. <https://doi.org/10.1016/J.ENVEXPBOT.2016.09.009> |
| 14. | Kihara S, Yamamoto K, Hisatomi A, Shiwa Y, Chu CC, Takada K, Ouyabe M, Pachakkil B, Kikuno H, Tanaka N, Shiwachi H (2022) Bacterial community of water yam (*Dioscorea alata* L.) cv. A-19. Microbes Environ 37(2):ME21062. doi: 10.1264/jsme2.ME21062 |
| 15. | Liu Y, Ponpandian LN, Kim H, Jeon J, Hwang BS, Lee SK, Park SC, Bae H (2019) Distribution and diversity of bacterial endophytes from four Pinus species and their efficacy as biocontrol agents for devastating pine wood nematodes. Sci Rep 9(1):12461. https://doi.org/10.1038/s41598-019-48739-4 |
| 16. | Nakkeeran S, Rajamanickam S, Saravanan R, Vanthana M, Soorianathasundaram K (2021) Bacterial endophytome-mediated resistance in banana for the management of *Fusarium* wilt. 3 Biotech 11(6):267. <https://doi.org/10.1007/s13205-021-02833-5> |
| 17. | Perazzolli M, Vicelli B, Antonielli L, Longa CMO, Bozza E, Bertini L, Caruso C, Pertot I (2022) Simulated global warming affects endophytic bacterial and fungal communities of Antarctic pearlwort leaves and some bacterial isolates support plant growth at low temperatures. Sci Rep. 12(1):18839. https://doi.org/https://doi.org/10.1038/s41598-022-23582-2 |
| 18. | Samuel SO, Suzuki K, Asiloglu R, Harada N (2022) Soil-root interface influences the assembly of the endophytic bacterial community in rice plants. Biol Fertil Soils 58(1):35-48. https://doi.org/10.1007/s00374-021-01611-y |
| 19. | Singh P, Singh RK, Guo D-J, Sharma A, Singh RN, Li D-P, Malviya MK, Song X-P, Lakshmanan P, Yang L-T, Li Y-R (2021) Whole genome analysis of sugarcane root-associated endophyte *Pseudomonas aeruginosa* B18—A plant growth-promoting bacterium with antagonistic potential against *Sporisorium scitamineum*. Front Microbiol 12:628376. https://doi.org/10.3389/fmicb.2021.628376 |
| 20. | Sun J, Chang M, Li H, Zhang Z, Chen Q, Chen Y, Yao Y, Pan A, Shi C, Wang C, Zhao J, Wan X (2019) Endophytic bacteria as contributors to theanine production in *Camellia sinensis*. J Agric Food Chem 67(38):10685-10693. https://doi.org/10.1021/acs.jafc.9b03946 |
| 21. | Um S, Lee J, Kim SH (2022) Lobophorin producing endophytic *Streptomyces olivaceus* JB1 Associated with *Maesa japonica* (Thunb.) Moritzi & Zoll. Front Microbiol 13:881253. https://doi.org/10.3389/fmicb.2022.881253 |
| 22. | Walitang DI, Kim K, Madhaiyan M, Kim YK, Kang Y, Sa T (2017) Characterizing endophytic competence and plant growth promotion of bacterial endophytes inhabiting the seed endosphere of Rice. BMC Microbiol17(1):209. https://doi.org/10.1186/s12866-017-1117-0 |
| 23. | Wang H, Wang Y, Jiang D, Xiang Z, Wang S, Kang C, [Zhang](https://scholar.google.com.mx/citations?user=BPZjRDcAAAAJ&hl=en&oi=sra) W, Ge Y, Wang T, Huang L, Liu D, Guo L (2022) Soil microbe inoculation alters the bacterial communities and promotes root growth of *Atractylodes lancea* under heat stress. Plant Soil 478:371-389. <https://doi.org/10.1007/s11104-022-05369-6> |
| 24. | Wang Q, Ge C, Xu S, Wu Y, Sahito ZA, Ma L, Pan F, Zhou Q, Huang L, Feng Y, Yang X (2020) The endophytic bacterium *Sphingomonas* SaMR12 alleviates Cd stress in oilseed rape through regulation of the GSH-AsA cycle and antioxidative enzymes. BMC Plant Biol 20(1):63. https://doi.org/10.1186/s12870-020-2273-1 |
| 25. | Worsley SF, Newitt J, Rassbach J, Batey SFD, Holmes NA, Murrell JC, Wilkinson B, Hutchings MI (2020) *Streptomyces* endophytes promote host health and enhance growth across plant species. Appl Environ Microbiol 86(16):e01053-20. https://doi.org/10.1128/AEM.01053-20 |
| 26. | Woźniak M, Gałązka A, Tyśkiewicz R, Jaroszuk-Ściseł J (2019) Endophytic bacteria potentially promote plant growth by synthesizing different metabolites and their phenotypic/physiological profiles in the Biolog GEN III MicroPlateTM t. Int J Mol Sci 20(21):5283. https://doi.org/10.3390/ijms20215283 |
| 27. | Zhang Y, Hua Q, Xu W, Mei L, Hu J, Zhang Z (2022) Response of root endosphere bacterial communities of typical rice cultivars to nitrogen fertilizer reduction at the jointing stage. Arch Microbiol 204(12):722. https://doi.org/10.1007/s00203-022-03334-6 |
| 28 | Zhang Z, Sun F, Chen Y, Yao L, Chen Z, Tian W (2019) *Mucilaginibacter endophyticus* sp. nov., an endophytic polysaccharide-producing bacterium isolated from a stem of *Miscanthus sinensis*. Antonie Van Leeuwenhoek 112(7):1087-1094. https://doi.org/10.1007/s10482-019-01242-2 |
