# Supplementary material for: Spatial and Temporal Shifts of Endophytic Bacteria in Conifer Seedlings of Abies religiosa (Kunth) Schltdl. & Cham
Source: Microb Ecol. 2024 Jul 3;87(1):90. doi: 10.1007/s00248-024-02398-9 (PMC11222277; doi:10.1007/s00248-024-02398-9)
Supplement: Supplementary file 1 — Supplementary file1 (PDF 2529 KB) [file 248_2024_2398_MOESM1_ESM.pdf]

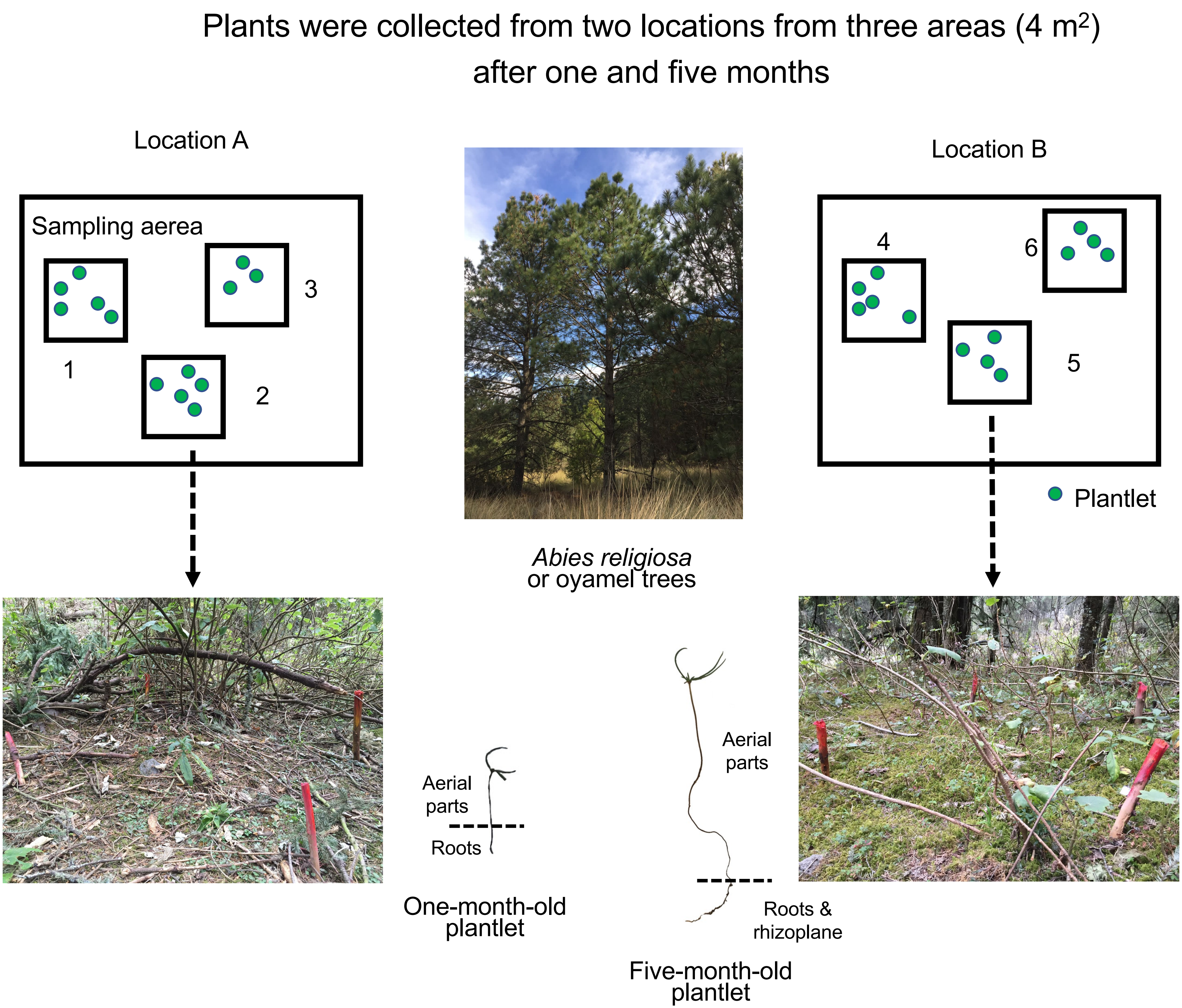
Plants were collected from two locations from three areas (4 m2)
after one and five months
Location A
Sampling aerea
1
2
3
Location B
4
5
6
Plantlet
Abies religiosa
or oyamel trees
Aerial
parts
Roots
One-month-old
plantlet
Aerial
parts
Roots &
rhizoplane
Five-month-old
plantlet
