# Supplementary material for: Spatial and Temporal Shifts of Endophytic Bacteria in Conifer Seedlings of Abies religiosa (Kunth) Schltdl. & Cham
Source: Microb Ecol. 2024 Jul 3;87(1):90. doi: 10.1007/s00248-024-02398-9 (PMC11222277; doi:10.1007/s00248-024-02398-9)
Supplement: Supplementary file 3 — Supplementary file3 (PDF 616 KB) [file 248_2024_2398_MOESM3_ESM.pdf]

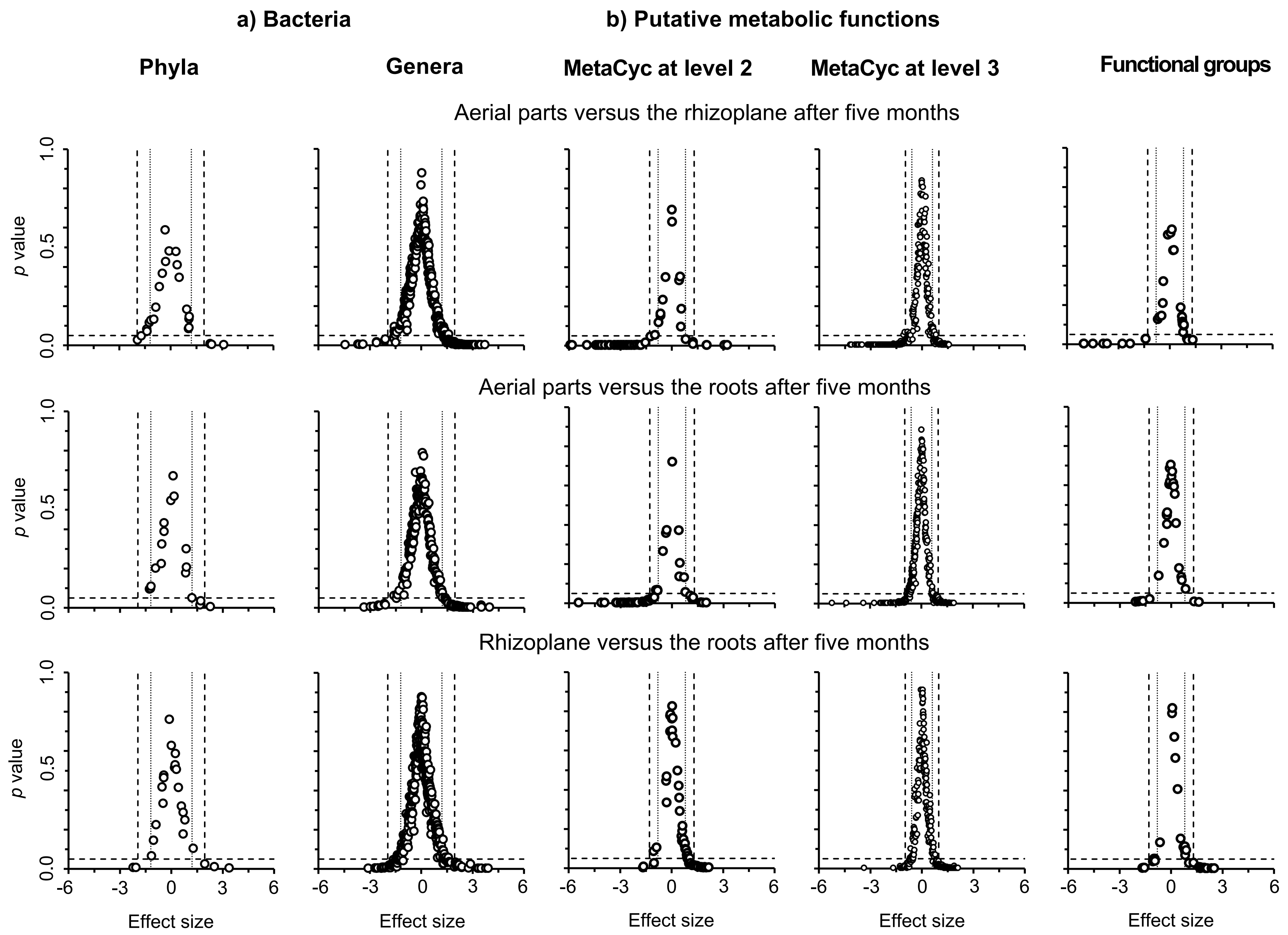
a) Bacteria
b) Putative metabolic functions
Phyla
Genera
MetaCyc at level 2
MetaCyc at level 3
Functional groups
Aerial parts versus the rhizoplane after five months
Aerial parts versus the roots after five months
Rhizoplane versus the roots after five months
p value
0.0
0.5
1.0
Effect size
-6
-3
0
3
6
