# Supplementary material for: Spatial and Temporal Shifts of Endophytic Bacteria in Conifer Seedlings of Abies religiosa (Kunth) Schltdl. & Cham
Source: Microb Ecol. 2024 Jul 3;87(1):90. doi: 10.1007/s00248-024-02398-9 (PMC11222277; doi:10.1007/s00248-024-02398-9)
Supplement: Supplementary file 4 — Supplementary file4 (PDF 479 KB) [file 248_2024_2398_MOESM4_ESM.pdf]

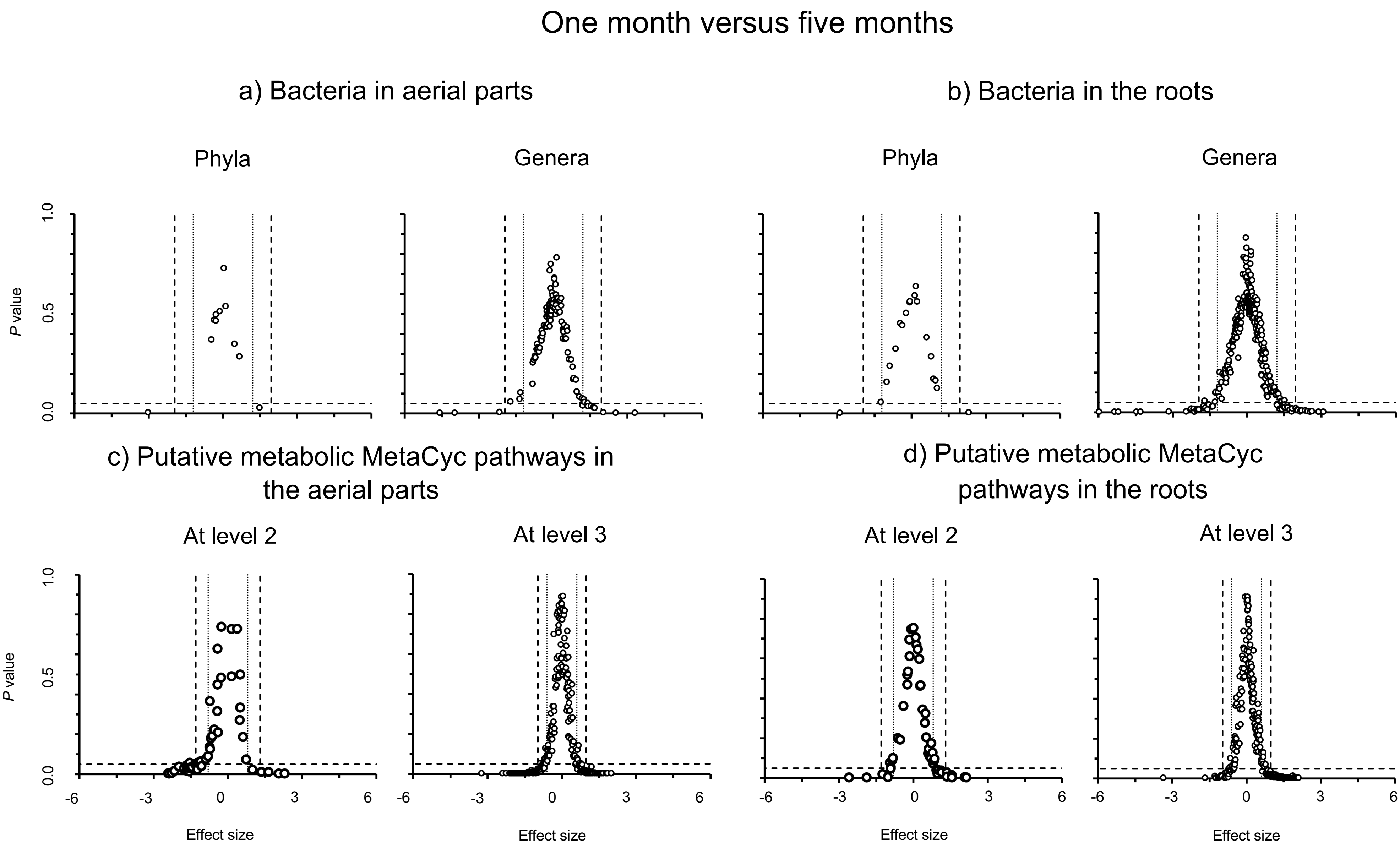
One month versus five months
a) Bacteria in aerial parts
b) Bacteria in the roots
Phyla
Genera
Phyla
Genera
c) Putative metabolic MetaCyc pathways in the aerial parts
d) Putative metabolic MetaCyc pathways in the roots
At level 2
At level 3
At level 2
At level 3
P value
0.0
0.5
1.0
-6
-3
0
3
6
Effect size
