# Supplementary material for: Spatial and Temporal Shifts of Endophytic Bacteria in Conifer Seedlings of Abies religiosa (Kunth) Schltdl. & Cham
Source: Microb Ecol. 2024 Jul 3;87(1):90. doi: 10.1007/s00248-024-02398-9 (PMC11222277; doi:10.1007/s00248-024-02398-9)
Supplement: Supplementary file 6 — Supplementary file6 (DOCX 27 KB) [file 248_2024_2398_MOESM6_ESM.docx]

**Table S2** Effect of plant parts (i.e., rhizoplane, roots and aerial parts) on the relative frequencies of bacterial phyla and genera, and putative metabolic pathways at level 3 of MetaCyc or putative functional groups after one or five months.

| ———————————————————————————————————————————————————————— |
| --- |
| Comparing the relative frequency of the bacterial groups in the aerial parts versus the roots after one month. A negative effect size means that the relative frequency was higher in the aerial parts than in the roots, while a positive value is the opposite |
| ———————————————————————————————————————————————————————— |
| Bacterial phyla ^a^: Actinomycetota (1.5 ^b^, 0.004 ^c^)  Bacterial genera: *Labrys* (1.4, 0.008), *Allorhizobium*-*Neorhizobium*-*Pararhizobium*-*Rhizobium* (1.4, 0.008), *Chitinophaga* (1.5, 0.007), *Mycobacterium* (1.5, 0.009), *Variovorax* (1.5, 0.004), *Streptomyces* (2.5, 0.004), *Mucilaginibacter* (2.5, 0.004)  MetaCyc pathways at level 3 ^d^: Glyoxylate cycle (-3.0, 0.004), Respiration (-2.9, 0.005), Carbohydrate Biosynthesis (-2.8, 0.004), Cell Structure Biosynthesis (-2.8, 0.004), Aminoacyl tRNA Charging (-2.7, 0.004), 1,5-anhydrofructose degradation (1.8, 0.004), Methylaspartate cycle (2.4, 0.004)  FAPROTAX ^e^: Chemoheterotrophy (-0.9, 0.056), Aerobic chemoheterotrophy (-0.8, 0.055), Nitrogen fixation (0.8, 0.115), Ureolysis (0.8, 0.083), Aromatic compound degradation (1.0, 0.043) |
| ———————————————————————————————————————————————————————— |
| Comparing the relative frequency of the bacterial groups in the aerial parts versus the rhizoplane after five months. A negative effect size means that the relative frequency was higher in the rhizoplane than in aerial parts, while a positive value is the opposite |
| ———————————————————————————————————————————————————————— |
| Bacterial phyla: Acidobacteriota (1.5, 0.010), Patescibacteria (1.5, 0.008), Planctomycetota (1.6, 0.005), Verrucomicrobiota (2.1, 0.004)  Bacterial genera: *Stenotrophomonas* (-3.0, 0.004), *Blastomonas* (-2.5, 0.007), *Acinetobacter* (-2.4, 0.006), *Novosphingobium* (-2.3, 0.007), *Taibaiella* (1.4, 0.009), *Mycobacterium* (1.4, 0.006), *Granulicella* (1.5, 0.009), *Dokdonella* (1.5, 0.009), *Labrys* (1.5, 0.006), Nitrosomonadaceae-*IS.44* (1.6, 0.008), Gammaproteobacteria-*WD260* (1.6, 0.006), *Roseiarcus* (1.6, 0.005), Rickettsiales-*SM2D12* (1.6, 0.005), Planctomycetota-*WD2101* soil group (1.7, 0.005), Nitrosomonadaceae-*Ellin6067* (1.8, 0.004), *Acidibacter* (1.8, 0.004), *Phenylobacterium* (1.8, 0.004), *Lacunisphaera* (1.8, 0.005), *Ferruginibacter* (1.9, 0.005), *Candidatus* Solibacter (1.9, 0.005), *Edaphobaculum* (1.9, 0.005), *Inquilinus* (2.0, 0.004), *Bryobacter* (2.0, 0.004), *Dongia* (2.1, 0.004), *Candidatus* Xiphinematobacter (2.1, 0.004), *Chthoniobacter* (2.2, 0.004), *Reyranella* (2.3, 0.004), *Mucilaginibacter* (2.5, 0.004)  MetaCyc pathways at level 3: Amino acid degradation (-5.9, 0.004), Carbohydrate degradation (-5.8, 0.004), Amine and polyamine biosynthesis (-5.0, 0.004), Glyoxylate cycle (-4.4, 0.004), TCA cycle (-4.3, 0.004), Methylaspartate cycle (2.0, 0.004), Chlorinated compound degradation (2.0, 0.004), Adenosylcobalamin biosynthesis II aerobic (3.2, 0.004)  FAPROTAX: Nitrate reduction (-4.5, 0.004), Nitrate respiration (-4.0, 0.004), Nitrogen respiration (-3.8, 0.004), Aromatic compound degradation (-2.9, 0.004), Aerobic chemoheterotrophy (-1.4, 0.028), Chemoheterotrophy (-1.4, 0.028), Phototrophy (1.2, 0.015), Photoheterotrophy (1.2, 0.020) |
| ———————————————————————————————————————————————————————— |

**Table S2** Continued

| ———————————————————————————————————————————————————————— |
| --- |
| Comparing the relative frequency of the bacterial groups in the aerial parts versus the roots after five months. A negative effect size means that the relative frequency was higher in the roots than in the aerial parts, while a positive value is the opposite |
| ———————————————————————————————————————————————————————— |
| Bacterial phyla: Verrucomicrobiota (1.5, 0.006)  Bacterial genera: *Novosphingobium* (-2.2, 0.005), *Pseudomonas* (-2.0, 0.007), *Haliangium* (1.4, 0.007), *Phenylobacterium* (1.4, 0.007), *Puia* (1.6, 0.004), *Dongia* (1.7, 0.005), *Mucilaginibacter* (1.8, 0.004), *Streptomyces* (2.3, 0.004)  MetaCyc pathways at level 3: Amino acid degradation (-5.4, 0.004), Carbohydrate degradation (-4.1, 0.004), Carboxylate degradation (-3.9, 0.004), superpathway of Glycolysis pyruvate dehydrogenase TCA and glyoxylate bypass (-3.3, 0.004), TCA cycle (-3.2, 0.004), Alcohol degradation (1.7, 0.006), Chlorinated compound degradation (1.8, 0.004),  FAPROTAX: Nitrogen respiration (-1.9, 0.009), Nitrate respiration (-1.9, 0.008), Nitrate reduction (-1.6, 0.015), Cellulolysis (0.8, 0.076) |
| ———————————————————————————————————————————————————————— |
| Comparing the relative frequency of the bacterial groups in the rhizoplane versus the roots after five months. A negative effect size means that the relative frequency was higher in the roots than in the rhizoplane, while a positive value the opposite. |
| ———————————————————————————————————————————————————————— |
| Bacterial phyla: Planctomycetota (-1.5, 0.008), Gemmatimonadota (-1.4, 0.009), Actinomycetota (2.2, 0.006)  Bacterial genera: *Candidatus* Udaeobacter (-1.8, 0.004), Burkholderiales-*TRA3.20* (-1.7, 0.005), *Rhodoplanes* (-1.7, 0.006), Gammaproteobacteria-*JG36.TzT.191* (-1.6, 0.006), Sutterellaceae-*AAP99* (1.8, 0.005), *Blastomonas* (2.2, 0.004), *Stenotrophomonas* (2.3, 0.004),  MetaCyc pathways at level 3: Adenosylcobalamin biosynthesis II (-1.7, 0.004), Superpathway of fucose and rhamnose degradation (-1.6, 0.010), Fatty Acid and lipid degradation (1.8, 0.004), Alcohol degradation (1.9, 0.006), Aldehyde degradation (2.0, 0.006), Aromatic compound degradation (2.0, 0.005), Carbohydrate degradation (2.2, 0.005)  FAPROTAX: Photoautotrophy (-1.8, 0.006), Anoxygenic photoautotrophy S oxidizing (-1.7, 0.006), Anoxygenic photoautotrophy (-1.5, 0.006), Nitrite denitrification (-1.0, 0.037), Nitrate denitrification (-1.0, 0.042), Nitrous oxide denitrification (-1.0, 0.043), photoheterotrophy (-0.9, 0.054) Denitrification (-0.9, 0.042), Phototrophy (-0.9, 0.052), Methylotrophy (0.8, 0.104), Dark hydrogen oxidation (0.8, 0.127), Methanol oxidation (0.8, 0.096), Aliphatic non methane hydrocarbon degradation (0.8, 0.070), Nitrogen fixation (1.2, 0.033), Aerobic chemoheterotrophy (1.6, 0.010), Chemoheterotrophy (1.6, 0.010), Aromatic compound degradation (1.8, 0.006), Nitrate respiration (1.8, 0.007), Nitrogen respiration (1.8, 0.006), Ureolysis (1.9, 0.004), Nitrogen reduction (2.1, 0.004) |
| ———————————————————————————————————————————————————————— |

**Table S2** Continued

| ———————————————————————————————————————————————————————— |
| --- |
| Comparing the relative frequency of the bacterial groups in the aerial parts after one month with those after five months. A negative effect size means that the relative frequency was higher in the aerial parts after one month than after five months, while a positive value the opposite |
| ———————————————————————————————————————————————————————— |
| Bacterial phyla: Pseudomonadota (-2.0, 0.007)  Bacterial genera: *Pseudomonas* (-2.7, 0.004), *Acinetobacter* (1.7, 0.005)  MetaCyc pathways at level 3: Polymeric compound degradation (-2.4, 0.005), Other degradation utilization (-2.4, 0.004), Glycan biosynthesis (-2.3, 0.006), Aldehyde degradation (2.1, 0.004)  FAPROTAX: Chemoheterotrophy (-2.5, 0.004), Aerobic chemoheterotrophy (-2.5, 0.004), Aromatic compound degradation (1.5, 0.011) |
| ———————————————————————————————————————————————————————— |
| Comparing the relative frequency of the bacterial groups in the roots after five months with those after one month. A negative effect size means that the relative frequency was higher in the roots after one month than after five months, while a positive value the opposite |
| ———————————————————————————————————————————————————————— |
| Bacterial phyla: Pseudomonadota (-1.9, 0.005), Myxococcota (1.5, 0.008)  Bacterial genera: *Pseudomonas* (-3.5, 0.004), *Novosphingobium* (-3.0, 0.004), *Methylobacterium-Methylorubrum* (-2.9, 0.004), *Pedobacter* (-2.1, 0.005), *Serratia* (-1.6, 0.006), *Robbsia* (1.5, 0.015), *Patulibacter* (-1.5, 0.017), *Chryseobacterium* (1.4, 0.019), *Flavobacterium* (-1.4, 0.005), *Devosia* (1.4, 0.012), *Bacillus* (-1.4, 0.012), *Pajaroellobacter* (1.4, 0.012), *Haliangium* (1.4, 0.005), *Puia* (1.7, 0.005), *Inquilinus* (1.7, 0.007)  MetaCyc pathways at level 3: Superpathway of fucose and rhamnose degradation (-2.6, 0.004), Superpathway of chorismate metabolism (-1.9, 0.004)  FAPROTAX: Chemoheterotrophy (-1.2, 0.032), Aerobic chemoheterotrophy (-1.2, 0.033), Cellulolysis (1.2, 0.027) |
| ———————————————————————————————————————————————————————— |

^a^ Only bacterial groups with an effect size ≥ 1.4 or ≤ -1.4 and a highly significant effect *p*<0.01 are given, ^b^ The effect size, which is defined as the difference between groups divided by the maximum dispersion within group A or B, was calculated with the aldex.ttest argument (ALDEx2 (version, 1.18), Gloor et al. (2020)), ^c^ non-parametric Kruskal Wallis test was used to calculate the *p* value with the aldex.kw argument (ALDEx2 (version, 1.18), Gloor et al. (2020)), ^d^ Only the functions with the five highest, and very large effect sizes (≥ 1.4 or ≤ -1.4) and highly significant effect *p*<0.01 are given (Kim, 2015), ^e^ Only functional groups with FAPROTAX with a large effect size ≥ 0.8 or ≤ -0.8 are given.

| ———————————————————————————————————————————————————————— |
| --- |
