# Supplementary material for: Spatial and Temporal Shifts of Endophytic Bacteria in Conifer Seedlings of Abies religiosa (Kunth) Schltdl. & Cham
Source: Microb Ecol. 2024 Jul 3;87(1):90. doi: 10.1007/s00248-024-02398-9 (PMC11222277; doi:10.1007/s00248-024-02398-9)
Supplement: Supplementary file 8 — Supplementary file8 (DOCX 22 KB) [file 248_2024_2398_MOESM8_ESM.docx]

**Table S4** Partitioning of beta diversity between the plant parts (rhizoplane, roots and aerial parts) and over time (one month versus five months) as calculated with the betapart package in R using the index.family “jaccard” (Baselga and Orme, 2012) of the amplicon sequence variants (ASVs) and putative metabolic pathways at level 3 of MetaCyc as determined with PICRUSt2

| ⎯⎯⎯⎯⎯⎯⎯⎯⎯⎯⎯⎯⎯⎯⎯⎯⎯⎯⎯⎯⎯⎯⎯⎯⎯⎯⎯⎯⎯⎯⎯⎯⎯⎯⎯⎯⎯⎯⎯⎯ | | | | |
| --- | --- | --- | --- | --- |
|  | index.family = "jaccard" | | | |
|  | ⎯⎯⎯⎯⎯⎯⎯⎯⎯⎯⎯⎯⎯⎯⎯⎯⎯⎯⎯ | | | |
|  | Analysis | beta.jtu ^a^ | beta.jne ^b^ | beta.jac ^c^ |
| ⎯⎯⎯⎯⎯⎯⎯⎯⎯⎯⎯⎯⎯⎯⎯⎯⎯⎯⎯⎯⎯⎯⎯⎯⎯⎯⎯⎯⎯⎯⎯⎯⎯⎯⎯⎯⎯⎯⎯⎯ | | | | |
|  | Bacterial amplificon sequence variants (ASVs) | | | |
| ⎯⎯⎯⎯⎯⎯⎯⎯⎯⎯⎯⎯⎯⎯⎯⎯⎯⎯⎯⎯⎯⎯⎯⎯⎯⎯⎯⎯⎯⎯⎯⎯⎯⎯⎯⎯⎯⎯⎯⎯ | | | | |
| Aerial parts vs roots after one month | beta.pair | 0.234 | 0.586 | 0.820 |
| Aerial parts, roots vs rhizoplane after five months | beta.multi | 0.601 | 0.283 | 0.884 |
| Aerial parts: One month vs five months | beta.temp | 0.605 | 0.010 | 0.615 |
| Roots: one month vs five months | beta.temp | 0.754 | 0.008 | 0.762 |
| ⎯⎯⎯⎯⎯⎯⎯⎯⎯⎯⎯⎯⎯⎯⎯⎯⎯⎯⎯⎯⎯⎯⎯⎯⎯⎯⎯⎯⎯⎯⎯⎯⎯⎯⎯⎯⎯⎯⎯⎯ | | | | |
|  | Putative metabolic pathways at level 3 of MetaCyc | | | |
| ⎯⎯⎯⎯⎯⎯⎯⎯⎯⎯⎯⎯⎯⎯⎯⎯⎯⎯⎯⎯⎯⎯⎯⎯⎯⎯⎯⎯⎯⎯⎯⎯⎯⎯⎯⎯⎯⎯⎯⎯ | | | | |
| Aerial parts vs roots after one month | beta.pair | 0.000 | 0.063 | 0.063 |
| Aerial parts, roots vs rhizoplane after five months | beta.multi | 0.020 | 0.039 | 0. 059 |
| Aerial parts: One month vs five months | beta.temp | 0.068 | 0.010 | 0.078 |
| Roots: one month vs five months | beta.temp | 0.035 | 0.005 | 0.040 |
| ⎯⎯⎯⎯⎯⎯⎯⎯⎯⎯⎯⎯⎯⎯⎯⎯⎯⎯⎯⎯⎯⎯⎯⎯⎯⎯⎯⎯⎯⎯⎯⎯⎯⎯⎯⎯⎯⎯⎯⎯ | | | | |

^a^ beta.jtu: dist dissimilarity matrix accounting for spatial turnover, measured as the turnover-fraction of Jaccard pair-wise dissimilarity, i.e. indicates 1-for-1 species substitutions, ^b^ beta.jne: dist object, dissimilarity matrix accounting for nestedness-resultant dissimilarity, measured as the nestedness-fraction of Jaccard pair-wise dissimilarity, i.e. indicates species gain or loss without substitution, ^c^ beta.jac: dist object, dissimilarity matrix accounting for beta diversity, measured as Jaccard pair-wise dissimilarity (a monotonic transformation of beta diversity), i.e. the full Jaccard index with values closer to 1 indicate greater dissimilarity (Baselga and Orme, 2012).
